# Thinner inner retinal layers are associated with lower cognitive performance, lower brain volume, and altered white matter network structure—The Maastricht Study

**DOI:** 10.1002/alz.13442

**Published:** 2023-08-23

**Authors:** Frank C. T. van der Heide, Indra L. M. Steens, Betsie Limmen, Sara Mokhtar, Martin P. J. van Boxtel, Miranda T. Schram, Sebastian Köhler, Abraham A. Kroon, Carla J. H. van der Kallen, Pieter C. Dagnelie, Martien C. J. M. van Dongen, Simone J. P. M. Eussen, Tos T. J. M. Berendschot, Carroll A. B. Webers, Marleen M. J. van Greevenbroek, Annemarie Koster, Thomas T. van Sloten, Jacobus F. A. Jansen, Walter H. Backes, Coen D. A. Stehouwer

**Affiliations:** ^1^ CARIM School for Cardiovascular Diseases Maastricht University (UM) Maastricht The Netherlands; ^2^ Department of Internal Medicine Maastricht University Medical Center+ (MUMC+) Maastricht The Netherlands; ^3^ Department of Psychiatry and Neuropsychology MUMC+ Maastricht MD The Netherlands; ^4^ School of Mental Health and Neuroscience MUMC+ Maastricht MD The Netherlands; ^5^ Heart and Vascular Center MUMC+ Maastricht The Netherlands; ^6^ CAPHRI Care and Public Health Research Institute UM MD The Netherlands; ^7^ Department of Epidemiology UM Maastricht The Netherlands; ^8^ University Eye Clinic Maastricht MUMC+ Maastricht The Netherlands; ^9^ Department of Social Medicine UM Maastricht The Netherlands; ^10^ Department of Vascular Medicine University Medical Centre Utrecht Utrecht The Netherlands; ^11^ Department of Radiology and Nuclear Medicine Maastricht University Medical Centre+ Maastricht The Netherlands

**Keywords:** brain structural connectivity, brain volume, clustering coefficient, cognitive function, cognitive performance, global efficiency, graph theory, grey matter, local efficiency, magnetic resonance imaging (MRI), optical coherence tomography (OCT), retinal imaging, retinal neurodegeneration, white matter, whole brain node degree

## Abstract

**INTRODUCTION:**

The retina may provide non‐invasive, scalable biomarkers for monitoring cerebral neurodegeneration.

**METHODS:**

We used cross‐sectional data from The Maastricht study (*n* = 3436; mean age 59.3 years; 48% men; and 21% with type 2 diabetes [the latter oversampled by design]). We evaluated associations of retinal nerve fiber layer, ganglion cell layer, and inner plexiform layer thicknesses with cognitive performance and magnetic resonance imaging indices (global grey and white matter volume, hippocampal volume, whole brain node degree, global efficiency, clustering coefficient, and local efficiency).

**RESULTS:**

After adjustment, lower thicknesses of most inner retinal layers were significantly associated with worse cognitive performance, lower grey and white matter volume, lower hippocampal volume, and worse brain white matter network structure assessed from lower whole brain node degree, lower global efficiency, higher clustering coefficient, and higher local efficiency.

**DISCUSSION:**

The retina may provide biomarkers that are informative of cerebral neurodegenerative changes in the pathobiology of dementia.

## 1 BACKGROUND

Clinical dementia is preceded by cognitive decline, and cerebral neurodegeneration, including the generalized loss of cerebral neurons and the deterioration of brain white matter network structure.^1^, ^2^, ^3^ Mechanistically, dysfunction of the neurovascular coupling unit is thought to predispose cerebral neuronal cells to ischemia, which can lead to white matter lesions, cerebral neurodegeneration (ie, loss of total grey and white matter and deterioration of brain networks), and cognitive decline, all of which are common features of clinical dementia.^1^, ^3^ In addition, amyloid beta and tau can accumulate over time in neuronal tissue and are also considered important contributors to dysfunction of the neurovascular coupling unit and cerebral neurodegeneration.^1^ Deterioration of brain white matter network structures is thought to lead to an imbalance between long‐ (ie, global) and short‐range (ie, local) brain connections (ie, a loss of small‐world brain organization), which hampers the fast and metabolically efficient transfer of information in the brain.^4^

The retina, postulated as a window to the brain, may provide non‐invasive, and scalable biomarkers that are informative of neurodegenerative cerebral changes in the pathobiology of dementia. Biologically, the anatomy and physiology of neurons in the inner retina and the brain are similar.^5^ Indeed, extensive data from epidemiological studies have shown that lower thicknesses of inner retinal layers (ie, the retinal nerve fiber layer [RNFL], the ganglion cell layer [GCL], and the inner plexiform layer [IPL]), which reflect retinal neurodegeneration, are associated with incident dementia,^6^ cognitive decline,^6^, ^7^, ^8^ worse cognitive performance,^6^, ^8^, ^9^, ^10^, ^11^, ^12^, ^13^, ^14^ and lower total grey and white matter brain volume.^15^, ^16^, ^17^

At present no studies have reported how retinal neurodegeneration is associated with white matter network structure as quantified from indices of long‐ (ie, global) and short‐range (ie, local) brain connections.^5^ However, to be able move towards using retinal neurodegenerative changes as biomarkers for dementia in the clinic, it is important to investigate this.

In view of the above, we investigated in a normal‐aging population‐based cohort study the associations of inner retinal layer thicknesses with indices of degenerative brain changes implicated in the pathobiology of dementia, that is, global cognitive performance, total grey and white matter volume, and brain white matter network structure.

## 2 METHODS

Here we provide key information. More details are provided in the online Supplementary information.

### 2.1 Study population and design

We used data from The Maastricht Study, an observational, population‐based cohort study. The rationale and methodology have been described previously.^18^ In brief, the study focuses on the etiology, pathophysiology, complications, and comorbidities of type 2 diabetes and is characterized by an extensive phenotyping approach. Eligible for participation were all individuals aged between 40 and 75 years and living in the southern part of the Netherlands. Participants were recruited through mass media campaigns, as well as from municipal registries and the regional Diabetes Patient Registry via mailings. Recruitment was stratified according to known type 2 diabetes status, with an oversampling of individuals with type 2 diabetes, for reasons of efficiency. The present report includes data from *N* = 7689 participants, who completed the baseline survey between November 2010 and December 2017.

Magnetic resonance imaging (MRI) measurements were implemented from December 2013 and were presently available for a subset of participants. The baseline examinations of each participant were performed within a time window of 3 months. The study has been approved by the medical ethical committee of Maastricht University (NL31329.068.10) and the Minister of Health, Welfare and Sports of the Netherlands (Permit 131088‐105234‐PG). All participants gave written informed consent.

### 2.2 Assessment of retinal thickness indices

We assessed peripapillary RNFL thickness (pRNFL; μm) and the thicknesses of the macular RNFL (mRNFL; μm); the macular GCL (mGCL; μm), and the macular IPL (mIPL; μm) in both eyes with optical coherence tomography (OCT; Spectralis unit and Eye Explorer version 5.7.5.0 software; Heidelberg Engineering, Heidelberg, Germany). We assessed pRNFL thickness with a 3.45 mm diameter circular scan (12°, 768 voxels, 100 automatic real‐time tracking) centered on the optic nerve head. We assessed the central macular area (Early Treatment Diabetic Retinopathy Study sectors 1 to 5) using a fovea‐centered macular volume scan (73 sections, 60 μm). Information on the assessment of OCT images and quality is presented in the Supplementary information and in Figures S1–S3.

### 2.3 Assessment of global cognitive performance, mild cognitive impairment, and dementia

We assessed three domains of cognitive performance with a concise neuropsychological test battery, that is, memory, information processing speed, and executive function.^18^ We assessed memory with the Verbal Learning Test^19^; information processing speed with parts I and II of the Stroop Color‐Word Test, parts A and B of the Concept Shifting Test, and the Letter‐Digit Substitution Test^20^, ^21^; and executive function with part III of the Stroop Color‐Word Test and part C of the Concept Shifting Test.^22^ Next, we expressed results per domain as z‐scores and constructed a composite score for global cognitive performance. In addition, we determined the presence of mild cognitive impairment. Mild cognitive impairment was considered present if cognitive performance in any domain (memory, executive function, information processing speed) was ≤1.5 SD below the expected cognitive performance (based on age, sex, and education level of the participant).

Date of dementia diagnosis was determined from hospital records. Medical records of all participants from Maastricht University Medical Center+ who gave consent were manually checked to identify potential cases of dementia. To acquire valid dementia diagnosis data, all potential cases of dementia were verified by a geriatric specialist and classified according to the *Diagnostic and Statistical Manual of Mental Disorders*, Fourth Edition (DSM‐IV) criteria.^23^

**RESEARCH IN CONTEXT**

1. **Systematic review**: We searched PubMed up to November 2022 to identify scientific articles on the association of inner retinal layers with white matter network structure, as quantified from indices of long‐ (ie, global) and short‐range (ie, local) brain connections. No previous population‐based cohort studies have investigated this association. In addition, we searched for articles on the associations of inner retinal layer thickness with cognitive performance and brain volume. These associations have already been investigated in cohort studies.
2. **Interpretation**: Our findings show that lower thickness of inner retinal layers is associated with worse cognitive performance, lower grey and white matter volume, and structural white matter network changes. Therefore, this study demonstrated, using population‐based data, that prior to the onset of mild cognitive impairment and dementia, retinal biomarkers may already be informative of cerebral neurodegenerative changes in the pathobiology of dementia.
3. **Future directions**: Retinal imaging tools may provide scalable, non‐invasive, and inexpensive biomarkers for the clinic. Future studies should evaluate the clinical value of retinal imaging tools as tools for the risk stratification of individuals at risk for accelerated neurocognitive aging and dementia in combination with other potentially scalable biomarkers.

### 2.4 Assessment of MRI measures

We assessed total grey and white matter volume with T1‐weighted MRI (3‐T scanner; Magnetom Prismafit Syngo MR D13D; Siemens Healthcare, Erlangen, Germany).^24^ We automatically segmented brain volumes with the FreeSurfer software package (Martinos Center for Biomedical Imaging, Boston, USA). In addition, we also segmented brain regions implicated in the pathobiology of mild cognitive impairment and Alzheimer's disease, that is, hippocampal volume, thalamus volume, cingulate cortex surface area, corpus callosum volume, cerebellum volume, and uncinate fasciculus volume.^25^

We assessed brain network structure with diffusion‐weighted MRI and estimated two measures of global brain network structure (“structural connectivity,” ie, whole brain node degree and global efficiency) and two measures of local brain network structure (ie, cluster coefficient and local efficiency) using the Brain Connectivity Toolbox in MATLAB (The MathWorks, Natick, USA).^24^ Whole brain node degree quantifies the average number of edges connected to a node (unit: edges), where a node is defined as a grey matter region, and an edge is defined as a connection between two nodes (ie, white matter).^4^ Then, global efficiency is quantified as the average inverse shortest path length (unit: connections), where a path reflects the number of (white matter) connections required for communication between two brain regions.^4^ Next, the clustering coefficient (no unit) quantifies the extent to which nodes are connected with neighboring nodes and is calculated as the number of edges of a node available divided by the total number of possible edges.^4^ Then, local efficiency quantifies the inverse of the average shortest path length in node neighborhoods (unit: connections).^4^ A neighborhood consists of the sum of edges that are directly adjacent to a certain node and all indirect edges which connect to these directly adjacent edges.^4^ Last, we normalized the graph measures from randomly generated networks (*N* = 100).

### 2.5 Assessment of covariates

As described previously,^18^ we assessed educational level (low, intermediate, high), smoking status (never, former, current), alcohol consumption (none, low, high), and history of cardiovascular disease (yes/no) by questionnaire^26^; glucose metabolism status (normal glucose metabolism, prediabetes, type 2 diabetes, types of diabetes other than type 2) from fasting venous plasma glucose samples (mmol/L) and 2‐h post load glucose samples (mmol/L); total cholesterol/high‐density lipid (HDL) ratio from fasting venous plasma samples (no unit); antihypertensive and lipid‐lowering medication use (yes/no) as part of an interview; waist circumference (cm) and office blood pressure (mm Hg) during a physical examination; intraocular pressure (mm Hg) and spherical equivalent (dpt) with an automated noncontact tonometer and refractor (Tonoref II; Nidek, Gamagori, Japan). Spherical equivalent was defined as the mean spherical equivalent of both eyes or as the spherical equivalent of the eye for which data were available (99% of all participants had data on spherical equivalent available for both eyes).

### 2.6 Statistical analyses

We used linear regression analyses to study the associations of determinants (ie, pRNFL, mRNFL, mGCL, and mIPL thickness) with outcomes (global cognitive performance, total grey matter volume, total white matter volume, whole brain node degree, global efficiency, clustering coefficient, and local efficiency). We inverted retinal thickness indices (ie, multiplied by −1) so that we could express associations per standard deviation (SD) of lower retinal thickness (indicating more neurodegeneration).^6^, ^7^, ^27^ We expressed the results of all analyses as standardized regression coefficients (*β*) with corresponding 95% confidence intervals (CIs). However, and only for the associations of pRFNL and mRNFL thicknesses with global cognitive performance, we expressed the associations as high versus low RNFL thickness because pRFNL and mRNFL were upon visual inspection nonlinearly associated with global cognitive performance (Figure S4), as found previously.^7^ Low thickness was defined as the lowest quartile and high thickness was defined as the highest three quartiles combined.

To start, we analyzed crude associations. Then, in model 1 we adjusted for age, sex, glucose metabolism status (entered as dummies, ie, type 2 diabetes, or prediabetes, or other types of diabetes vs. normal glucose metabolism status [reference]), educational level (low [reference], middle, high), and spherical equivalent. Additionally, and only for analyses with outcomes estimated from MRI, we adjusted for MRI lag time in model 1 (median [interquartile range] lag time was 0.7 [0.3 to 1.1] years). Next, in model 2, we additionally adjusted for common risk factors for neurodegeneration, that is, office systolic blood pressure, history of cardiovascular disease, use of antihypertensive medication, waist circumference, total cholesterol/HDL cholesterol ratio, lipid‐modifying medication, smoking and alcohol consumption.^28^ We adjusted for covariates in model 2 in a separate model as these factors may be potential confounders and potential causes of neurodegeneration.^29^ Adjustment for potential causes of neurodegeneration may increase accuracy of the estimate.^29^

We tested for interaction by sex and glucose metabolism status to assess whether the associations under investigation differed in strength between men and women, respectively, or between individuals with type 2 diabetes, prediabetes, or normal glucose metabolism. To test for interaction, we entered interaction terms with the determinant and all covariates in the fully adjusted model (eg, sex*pRNFL thickness), as previously described.^30^ A statistically significant *P*‐value for an interaction indicates that the association under study differs between subgroups (ie, between men and women, or between individuals with type 2 diabetes or prediabetes vs. individuals with normal glucose metabolism). For interaction analyses with glucose metabolism status, we excluded participants with other types of diabetes from the interaction analyses because the number of these participants was small (*n* = 20).

### 2.7 Additional analyses

We performed a range of additional analyses. Details are provided in the online Supplementary information. First, we separately analyzed the associations of retinal thickness indices with individual regional brain structures implicated in the pathobiology of mild cognitive impairment and Alzheimer's disease, that is, hippocampal volume, thalamus volume, cingulate cortex surface area, corpus callosum volume, cerebellum volume, and uncinate fasciculus volume.^25^ Second, we analyzed the associations of retinal thickness indices with individual cognitive domains, that is, memory, executive function, and information processing speed. Third, we additionally adjusted for a range of covariates (ie, potential confounders) that were not included in the main analyses for reasons of missing data (eg, physical activity and dietary intake) or because these covariates may be confounders, potential mediators and/or descendants of the outcome (eg, the presence of a major depressive episode). Fourth, we excluded individuals with retinal diseases (ie, diabetic retinopathy, glaucoma, and age‐related macular degeneration). Last, we performed additional analyses in which we replaced waist circumference, glucose metabolism status, office systolic blood pressure, and educational level with other covariates that reflect similar underlying constructs.

We performed analyses in R (version 4.0.3 [2020‐10‐10], R Foundation for Statistical Computing, Vienna, Austria). For all analyses, including interaction analyses, a *P*‐value of <0.05 was considered statistically significant.

## 3 RESULTS

### 3.1 Selection and characteristics of the study population

**FIGURE 1:**
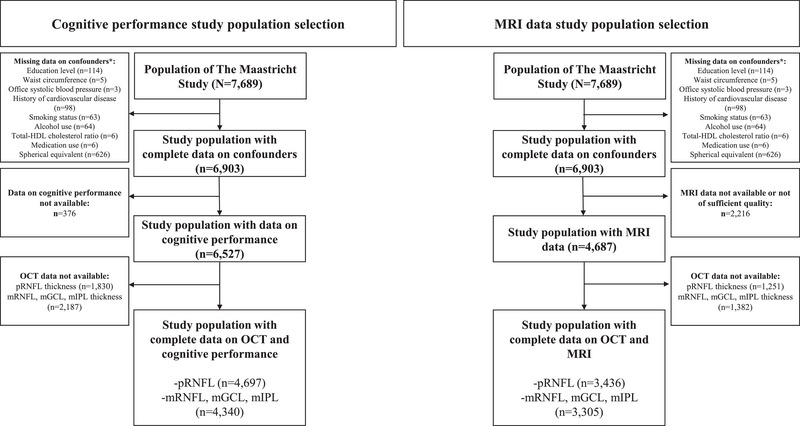
Study population selection. *Not mutually exclusive. HDL, high density lipoprotein; mGCL, macular ganglion cell layer; mIPL, macular inner plexiform layer; MRI, magnetic resonance imaging; mRNFL, macular retinal nerve fiber layer; OCT, optical coherence tomography; pRNFL, peripapillary retinal nerve fiber layer

Figure 1 presents an overview of the study population selection. Table 1 and Table S1 show general characteristics of the study population for pRNFL thickness and global cognitive performance. Overall, participants with a lower pRNFL thickness were older, were more frequently men, had a lower education level, and had a worse cardiovascular risk profile.

**TABLE 1:** General study population characteristics according to tertiles of pRNFL thickness in the study population with complete data on cognitive performance

	pRNFL				
		Low pRNFL thickness	Middle pRNFL thickness	High pRNFL thickness	
Characteristic	Overall, *N* = 4697	Tertile 1, *N* = 1566	Tertile 2, *N* = 1566	Tertile 3, *N* = 1565	*P*‐value
Age (years)	59.27 ± 8.68	59.85 ± 8.62	59.01 ± 8.70	58.95 ± 8.70	0.01
Sex					<0.001
Men	2257 (48)	855 (55)	710 (45)	692 (44)	
Women	2440 (52)	711 (45)	856 (55)	873 (56)	
Educational status					0.003
Low	1591 (34)	493 (31)	537 (34)	561 (36)	
Middle	1343 (29)	426 (27)	454 (29)	463 (30)	
High	1763 (38)	647 (41)	575 (37)	541 (35)	
Glucose metabolism status					<0.001
Normal	2957 (63)	915 (58)	1016 (65)	1026 (66)	
Prediabetes	712 (15)	240 (15)	242 (15)	230 (15)	
Type 2 diabetes	1008 (21)	404 (26)	301 (19)	303 (19)	
Other types of diabetes than type 2 diabetes	20 (0.4)	7 (0.4)	7 (0.4)	6 (0.4)	
Office systolic blood pressure (mmHg)	133.02 ± 17.63	134.40 ± 17.30	132.53 ± 17.55	132.13 ± 17.95	<0.001
Cardiovascular disease	766 (16)	273 (17)	249 (16)	244 (16)	0.32
Waist circumference (cm)	94.37 ± 13.28	95.44 ± 13.37	93.65 ± 12.87	94.04 ± 13.52	<0.001
Total/HDL cholesterol ratio	3.36 (2.75–4.17)	3.41 (2.78–4.17)	3.35 (2.76–4.22)	3.33 (2.71–4.15)	0.44
Use of lipid‐modifying medication (yes/no)	1416 (30)	517 (33)	442 (28)	457 (29)	0.01
Use of antihypertensive medication (yes/no)	1681 (36)	644 (41)	520 (33)	517 (33)	<0.001
Alcohol consumption					0.41
Low	830 (18)	258 (16)	292 (19)	280 (18)	
Middle	2743 (58)	920 (59)	896 (57)	927 (59)	
High	1124 (24)	388 (25)	378 (24)	358 (23)	
Smoking status					0.004
Never	1820 (39)	600 (38)	592 (38)	628 (40)	
Former	2293 (49)	793 (51)	790 (50)	710 (45)	
Current	584 (12)	173 (11)	184 (12)	227 (15)	
Spherical equivalent (dpt)	−0.27 ± 2.38	−1.23 ± 2.66	−0.19 ± 2.14	0.61 ± 1.93	<0.001
MRI lag time (years)^a^	0.69 (0.26–1.09)	0.77 (0.30–1.19)	0.70 (0.28–1.09)	0.60 (0.22–1.04)	<0.001
Determinants					
pRNFL (micrometer)	94.92 ± 10.89	83.26 ± 6.70	95.33 ± 2.55	106.17 ± 6.44	NA
mRNFL (micrometer)^b^	22.54 ± 4.30	20.07 ± 0.96	22.02 ± 0.47	25.53 ± 6.25	NA
mGCL(micrometer)^b^	43.99 ± 4.56	41.14 ± 4.35	44.32 ± 3.45	46.52 ± 4.10	NA
mIPL (micrometer)^b^	37.50 ± 3.19	35.66 ± 2.96	37.73 ± 2.53	39.12 ± 3.06	NA
Brain					
Cognitive performance (SD)	0.00 ± 1.00	0.01 ± 0.69	0.08 ± 0.64	0.04 ± 0.67	0.02
Mild cognitive impairment	1052 (22)	332 (21)	351 (22)	369 (24)	0.27
Dementia	3 (< 0.1)	1 (< 0.1)	0 (0)	2 (< 0.1)	0.37
Total grey matter brain volume (ml)^a^	47.73 ± 2.32	47.47 ± 2.40	47.87 ± 2.25	47.84 ± 2.29	<0.001
Total white matter brain volume (ml)^a^	34.20 ± 2.06	33.94 ± 2.07	34.16 ± 2.07	34.50 ± 1.99	<0.001
Whole brain node degree (edges)^a^	17.77 ± 0.34	17.72 ± 0.37	17.79 ± 0.32	17.81 ± 0.34	<0.001
Global efficiency (connections)^a^	0.84 ± 0.03	0.84 ± 0.03	0.84 ± 0.03	0.84 ± 0.03	0.47
Clustering coefficient (no unit)^a^	2.31 ± 0.08	2.32 ± 0.08	2.31 ± 0.07	2.30 ± 0.07	<0.001
Local efficiency (connections)^a^	1.49 ± 0.04	1.50 ± 0.04	1.49 ± 0.04	1.49 ± 0.04	<0.001

*Notes*: Data are presented as mean ± standard deviation, median (interquartile range), or number (%). *P*‐values were calculated using an analysis of variance test (ANOVA; for continuous variables with a normal distribution), a Mann‐Whitney test (for continuous variables without a normal distribution), or a Chi‐square test (for categorical variables).

Abbreviations: HDL, high‐density lipid; mGCL, macular ganglion cell layer; mIPL, macular inner plexiform layer; MRI, magnetic resonance imaging; mRNFL, macular retinal nerve fiber layer; NA, not applicable; pRNFL, peripapillary retinal nerve fiber layer; SD, standard deviation.

^a^ Data shown in the study population with complete data on pRNFL and MRI data (*n* = 3436).

^b^ Data shown in the study population with complete data on mRNFL, mGCL mIPL, and cognitive performance (*n* = 4340).

There were *n* = 3 (<0.1%) participants with dementia and *n* = 1052 (22%) participants with mild cognitive impairment. Table S2 shows retinal and brain metrics according to mild cognitive impairment status. General characteristics of the included participants were generally highly comparable to those of participants excluded due to missing data (Tables S3 and S4).

### 3.2 Associations with global cognitive performance

**FIGURE 2:**
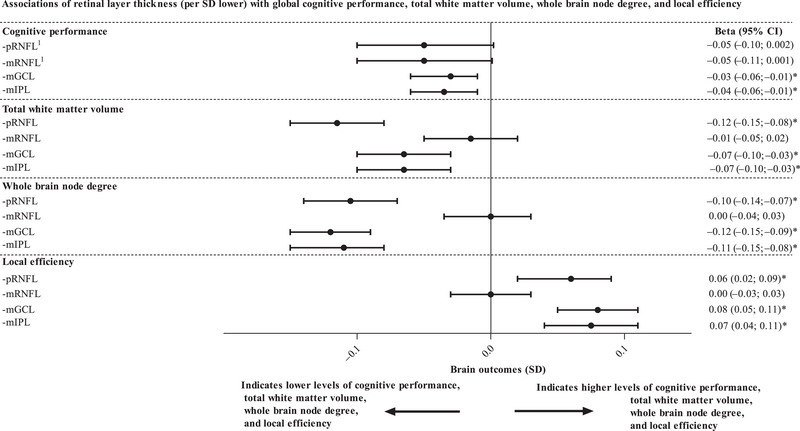
Associations of retinal layer thickness indices (per SD lower) with global cognitive performance, total white matter volume, whole brain node degree, and local efficiency. Regression coefficients (*β*) represent the difference in global cognitive performance, total white matter volume, whole brain node degree, and local efficiency (all expressed in SD) per SD lower pRNFL thickness, mRNFL thickness, mGCL thickness, and mIPL thickness. The associations of retinal layer thicknesses with total grey matter volume, global efficiency and clustering coefficient are not shown as results for these measures were similar to those shown in Figure 2 (the results for total grey matter volume were similar to the results for total white matter volume; the results for global efficiency were directionally similar to the results for whole brain node degree; and the results for clustering coefficient were directionally and numerically similar to the results for local efficiency; all shown in Tables 3 and 4). Values per SD or quartile of retinal thickness are reported in the legend of Table 2; values per SD of MRI measures are reported in the legend of Tables 3 and 4. Variables entered in the models in addition to retinal indices are age, sex, glucose metabolism status, educational level, spherical equivalent, MRI lag time (only applicable for MRI indices), office systolic blood pressure, history of cardiovascular disease, waist circumference, smoking status, alcohol consumption, total/HDL cholesterol ratio, lipid‐modifying medication, and antihypertensive medication. *Indicates statistically significant (*P* < 0.05). Superscript (^1^) indicates that the associations of indices of pRNFL and mRNFL with cognitive performance are shown for low versus high thickness instead of per SD lower. CI, confidence interval; HDL, high‐density lipid; mGCL, macular ganglion cell layer; mIPL, macular inner plexiform layer; MRI, magnetic resonance imaging; mRNFL, macular retinal nerve fiber layer; pRNFL, peripapillary retinal nerve fiber layer; SD, standard deviation

After full adjustment (model 2), lower mGCL and mIPL thicknesses were significantly associated with lower cognitive performance (*β* [95% CI], −0.03 [−0.06 to −0.01] and −0.04 [−0.06 to −0.01], respectively; Table 2 and Figure 2). Next, after full adjustment (model 2), low versus high pRNFL and mRNFL thicknesses were not significantly associated with lower cognitive performance (low vs. high thickness, cognitive performance in SD [95% CI], −0.05 [−0.10 to 0.002] and −0.05 [−0.11 to 0.002], respectively).

**TABLE 2:** Associations of retinal thickness indices with global cognitive performance

			Global cognitive performance	
Retinal thickness indices	Model	*N*	Beta (95% CI)	*P*‐value
Peripapillary retinal nerve fiber layer thickness, low versus high	Crude	4697	−0.11 (−0.17 to −0.04)	**0.002**
	1	4697	−0.05 (−0.10 to 0.002)	0.068
	2	4697	−0.05 (−0.10 to 0.002)	0.070
Macular retinal nerve fiber layer thickness, low versus high	Crude	4340	−**0.13 (−0.20 to −0.06)**	**<0.001**
	1	4340	−**0.06 (−0.12 to −0.01)**	**0.030**
	2	4340	−0.05 (−0.11 to 0.001)	0.055
Macular ganglion cell layer thickness, per SD lower	Crude	4340	−**0.14 (−0.17 to −0.11)**	**<0.001**
	1	4340	−**0.04 (−0.06 to −0.01)**	**0.002**
	2	4340	−**0.03 (−0.06 to −0.01)**	**0.005**
Macular inner plexiform layer thickness, per SD lower	Crude	4340	−**0.13 (−0.16 to −0.10)**	**<0.001**
	1	4340	−**0.03 (−0.04 to −0.01)**	**0.002**
	2	4340	−**0.04 (−0.06 to −0.01)**	**0.004**

*Notes*: Table 2 shows the associations of retinal thickness indices with cognitive performance. One SD corresponds with 10.89 micrometer peripapillary retinal nerve fiber layer thickness, 4.30 micrometer macular retinal nerve fiber layer thickness, 4.56 micrometer macular ganglion cell layer thickness and 3.19 micrometer macular inner plexiform layer thickness. Of note, nonlinear associations are shown for macular and peripapillary retinal nerve fiber layer thickness. For peripapillary RNFL thickness, the median [interquartile range] in the low (*n* = 1175) and high (*n* = 3522) thickness groups, respectively, are 82.95 [78.61 to 85.78] µm and 98.63 [93.72 to 104.03] µm. For macular RNFL thickness, the median [interquartile range] in the low (*n* = 1085) and high (*n* = 3255) thickness groups, respectively, are 19.98 [19.34 to 20.43] µm and 22.63 [21.70 to 23.88] µm. Variables entered in the models: Crude: none; Model 1: + age, sex, glucose metabolism status, educational level, spherical equivalent; Model 2: model 1 + office systolic blood pressure, history of cardiovascular disease, waist circumference, smoking status, alcohol consumption, Total/HDL cholesterol ratio, lipid‐modifying medication, and antihypertensive medication. Bold denotes *P* < 0.05.

Abbreviations: CI, confidence interval; HDL, high‐density lipid; N, population sample size; SD, standard deviation.

### 3.3 Associations with brain volume

After full adjustment (model 2), lower mGCL and mIPL thicknesses were significantly associated with lower total grey matter volume (0.05 [−0.08 to −0.02] and −0.05 [−0.08 to −0.02], respectively; Table 3 and Figure 2). Again, after full adjustment (model 2), lower pRNFL and mRNFL thicknesses were both not associated with total grey matter volume (0.00 [−0.03 to 0.03] and 0.00 [−0.03 to 0.03], respectively).

**TABLE 3:** Associations of retinal thickness indices with total grey matter brain volume and total white matter brain volume

			Total grey matter volume		Total white matter volume	
Retinal thickness indices	Model	Number of participants	Beta (95% CI)	*P*‐value	Beta (95% CI)	*P*‐value
Peripapillary retinal nerve fiber layer thickness, per SD lower	Crude	3436	**−0.07 (−0.10 to −0.04)**	**<0.001**	**−0.12 (−0.16 to −0.09)**	**<0.001**
	1	3436	0.00 (−0.03 to 0.03)	0.93	**−0.12 (−0.15 to −0.09)**	**<0.001**
	2	3436	0.00 (−0.03 to 0.03)	0.85	**−0.12 (−0.15 to −0.08)**	**<0.001**
Macular retinal nerve fiber layer thickness, per SD lower	Crude	3305	**0.06 (0.02 to 0.09)**	**<0.001**	0.00 (−0.03 to 0.03)	0.97
	1	3305	0.01 (−0.02 to 0.04)	0.74	−0.01 (−0.05 to 0.02)	0.41
	2	3305	0.00 (−0.03 to 0.03)	0.91	−0.01 (−0.05 to 0.02)	0.42
Macular ganglion cell layer thickness, per SD lower	Crude	3305	**−0.13 (−0.16 to −0.10)**	**<0.001**	**−0.14 (−0.17 to −0.10)**	**<0.001**
	1	3305	**−0.06 (−0.09 to −0.03)**	**<0.001**	**−0.07 (−0.10 to −0.04)**	**<0.001**
	2	3305	**−0.05 (−0.08 to −0.02)**	**0.001**	**−0.07 (−0.10 to −0.03)**	**<0.001**
Macular inner plexiform layer thickness, per SD lower	Crude	3305	**−0.12 (−0.15 to −0.08)**	**<0.001**	**−0.14 (−0.17 to −0.10)**	**<0.001**
	1	3305	**−0.05 (−0.08 to −0.02)**	**<0.001**	**−0.07 (−0.10 to −0.04)**	**<0.001**
	2	3305	**−0.05 (−0.08 to −0.02)**	**0.002**	**−0.07 (−0.10 to −0.03)**	**<0.001**

*Notes*: Table 3 shows the associations of retinal thickness indices with cognitive performance, total grey matter and white matter brain volume. Values per SD of retinal thickness are numerically similar to the values shown in the legend of Table 2. One SD of total grey and white matter volume, respectively, corresponds with 2.32 and 2.06 mL (calculated in the study population with complete data on peripapillary retinal nerve fiber layer thickness). Variables entered in the models: Crude: none; Model 1: + age, sex, glucose metabolism status, educational level, spherical equivalent, and MRI lag time; Model 2: model 1 + office systolic blood pressure, history of cardiovascular disease, waist circumference, smoking status, alcohol consumption, Total/HDL cholesterol ratio, lipid‐modifying medication, and antihypertensive medication. Bold denotes *P* < 0.05.

Abbreviations: CI, confidence interval; HDL, high‐density lipid; MRI, magnetic resonance imaging; N, population sample size; SD, standard deviation.

After full adjustment (model 2), lower pRNFL, mGCL, and mIPL thicknesses were significantly associated with lower total white matter volume (standardized betas [95% CI], −0.12 [−0.15 to −0.08], −0.07 [−0.10 to −0.03], and −0.07 [−0.10; −0.03], respectively; Table 3). Again, after full adjustment (model 2), lower mRNFL thickness was not associated with lower total white matter volume (−0.01 [−0.05 to 0.02]).

### 3.4 Associations with structural connectivity

After full adjustment (model 2), lower pRNFL, mGCL, and mIPL thicknesses were significantly associated with lower whole brain node degree (−0.10 [−0.14 to −0.07], −0.12 [−0.15 to −0.09], and −0.11 [−0.15 to −0.08], respectively; Table 4 and Figure 2). Next, after full adjustment (model 2), lower pRNFL, mGCL, and mIPL thicknesses were significantly associated with higher clustering coefficient (0.05 [0.02 to 0.09], 0.07 [0.04 to 0.11], and 0.07 [0.03 to 0.10], respectively) and higher local efficiency (0.06 [0.02 to 0.09], 0.08 [0.05 to 0.11], and 0.07 [0.04 to 0.11], respectively).

**TABLE 4:** Associations of retinal thickness indices with structural connectivity indices

			Whole brain node degree		Global efficiency		Clustering coefficient		Local efficiency	
Retinal thickness indices	Model	Number of participants	Beta (95% CI)	*P*‐value	Beta (95% CI)	*P*‐value	Beta (95% CI)	*P*‐value	Beta (95% CI)	*P*‐value
Peripapillary retinal nerve fiber layer thickness, per SD lower	Crude	3436	**−0.12 (−0.15 to −0.09)**	**<0.001**	**−0.03 (−0.06 to 0.00)**	0.085	**0.08 (0.05 to 0.11)**	**<0.001**	**0.08 (0.05 to 0.12)**	**<0.001**
	1	3436	**−0.11 (−0.14 to −0.07)**	**<0.001**	**−0.04 (−0.08 to −0.01)**	**0.020**	**0.05 (0.02 to 0.09)**	**0.002**	**0.06 (0.02 to 0.09)**	**<0.001**
	2	3436	**−0.10 (−0.14 to −0.07)**	**<0.001**	**−0.04 (−0.08 to −0.01)**	**0.017**	**0.05 (0.02 to 0.09)**	**0.003**	**0.06 (0.02 to 0.09)**	**<0.001**
Macular retinal nerve fiber layer thickness, per SD lower	Crude	3305	0.02 (−0.01 to 0.06)	0.22	−0.01 (−0.04 to 0.03)	0.64	−0.03 (−0.06 to 0.01)	0.10	−0.03 (−0.06 to 0.01)	0.10
	1	3305	0.00 (−0.03 to 0.03)	0.93	0.00 (−0.03 to 0.04)	0.93	0.00 (−0.03 to 0.03)	0.98	0.00 (−0.03 to 0.03)	0.93
	2	3305	0.00 (−0.04 to 0.03)	0.92	0.00 (−0.03 to 0.03)	>0.99	0.00 (−0.03 to 0.04)	0.89	0.00 (−0.03 to 0.03)	0.93
Macular ganglion cell layer thickness, per SD lower	Crude	3305	**−0.18 (−0.21 to −0.14)**	**<0.001**	0.00 (−0.03 to 0.03)	0.98	**0.11 (0.08 to 0.15)**	**<0.001**	**0.12 (0.09 to 0.15)**	**<0.001**
	1	3305	**−0.12 (−0.16 to −0.09)**	**<0.001**	−0.03 (−0.07 to 0.00)	0.052	**0.07 (0.04 to 0.11)**	**<0.001**	**0.08 (0.05 to 0.11)**	**<0.001**
	2	3305	**−0.12 (−0.15 to −0.09)**	**<0.001**	−0.03 (−0.07 to 0.00)	0.063	**0.07 (0.04 to 0.11)**	**<0.001**	**0.08 (0.05 to 0.11)**	**<0.001**
Macular inner plexiform layer thickness, per SD lower	Crude	3305	**−0.16 (−0.20 to −0.13)**	**<0.001**	0.01 (−0.03 to 0.04)	0.64	**0.10 (0.06 to 0.13)**	**<0.001**	**0.11 (0.07 to 0.14)**	**<0.001**
	1	3305	**−0.12 (−0.15 to −0.08)**	**<0.001**	−0.02 (−0.06 to 0.01)	0.17	**0.07 (0.03 to 0.10)**	**<0.001**	**0.07 (0.04 to 0.11)**	**<0.001**
	2	3305	**−0.11 (−0.15 to −0.08)**	**<0.001**	−0.02 (−0.06 to 0.01)	0.18	**0.07 (0.03 to 0.10)**	**<0.001**	**0.07 (0.04 to 0.11)**	**<0.001**

*Notes*: Table 4 shows the associations of retinal thickness indices with indices of structural connectivity. Values per SD of retinal thickness are numerically similar to the values presented in the legend of Table 2. Values per SD are 0.34 edges for whole brain node degree; 0.03 connections for global efficiency; 0.08 (no unit) for clustering coefficient; and 0.04 connections for local efficiency (calculated in the study population with complete data on peripapillary retinal nerve fiber layer thickness). Variables in the models: Model 1: + age, sex, glucose metabolism status, educational level, spherical equivalent, and MRI lag time; Model 2: model 1 + office systolic blood pressure, history of cardiovascular disease, waist circumference, smoking status, alcohol consumption, Total/HDL cholesterol ratio, lipid‐modifying medication, and antihypertensive medication. Bold denotes *P* < 0.05.

Abbreviations: CI, confidence interval; HDL, high‐density lipid; MRI, magnetic resonance imaging; N, population sample size; SD, standard deviation.

Similarly, after full adjustment (model 2), lower pRNFL thickness was significantly associated with lower global efficiency (−0.04 [−0.08 to −0.01]). Next, after full adjustment (model 2), lower mGCL and mIPL thicknesses were not significantly associated with lower global efficiency (−0.03 [−0.07 to 0.002] and −0.02 [−0.06 to 0.01], respectively).

Then, after full adjustment (model 2), lower mRNFL thickness was neither associated with whole brain node degree (0.00 [−0.04 to 0.03]), global efficiency (0.00 [−0.03 to 0.03]), clustering coefficient (0.00 [−0.03 to 0.04]), nor local efficiency (0.00 [−0.03 to 0.03]).

### 3.5 Interaction analyses

Overall, results of interaction analyses did not show a consistent pattern. This indicates that the strengths of the associations under study did not consistently differ between men and women, or between individuals with normal glucose metabolism status, prediabetes, or type 2 diabetes (all *P*‐values for interaction are shown in Table S5). The majority of associations of retinal metrics (*n* = 4) with brain metrics (*n* = 7) was not modified (we tested for interactions in 4*7 = 28 associations). Sex did not modify *n* = 26/28 associations, prediabetes did not modify *n* = 27/28 associations, and type 2 diabetes did not modify *n* = 27/28 associations.

### 3.6 Additional analyses

We observed numerically similar results in a range of additional analyses (Tables S5–S8; more details are presented in the Supplementary information, Supplemental Results section). First, we found that lower thicknesses of most inner retinal layers (ie, all layers except for mRNFL) were associated with lower brain volume or surface area of brain regions implicated in the pathobiology of Alzheimer's disease and mild cognitive impairment (ie, hippocampal volume, thalamus volume, cingulate cortex surface area, corpus callosum volume, cerebellum volume, and uncinate fasciculus volume; Table 5). Second, we had similar findings when we analyzed associations of thicknesses of inner retinal layers with individual cognitive domains as outcome instead of global cognitive performance (Table S5). Of note, associations were somewhat stronger for executive function and information processing speed than for memory. Third, we had similar findings to those shown in the main analyses when we performed additional analyses in which we adjusted for variables that were not included in the main analyses for reasons of missing data or because these covariates may be confounders, potential mediators, and/or descendants of the outcome (Tables S7 and S8). Fourth, we had similar findings when we when we excluded individuals with a retinal disease (ie, diabetic retinopathy, glaucoma, or age‐related macular degeneration; Table S7 and S8). However, and only when we excluded individuals with a retinal disease, we found that lower mRNFL thickness was significantly associated with lower total white matter volume (standardized beta [95% CI], −0.05 [−0.09 to −0.02]; Table S8). Last, we had similar findings when we replaced waist circumference, glucose metabolism status, office systolic blood pressure, and educational level with covariates that reflect similar underlying constructs (data not shown).

**TABLE 5:** Associations of retinal thickness indices with brain metrics of regions implicated in the pathobiology of mild cognitive impairment and Alzheimer's disease (ie, hippocampal volume, thalamus volume, cingulate cortex surface area, corpus callosum volume, cerebellum volume, and uncinate fasciculus volume)

Retinal thickness indices														
	Model	Number of participants	Hippocampal volume		Thalamus volume		Cingulate cortex surface area		Corpus callosum volume		Cerebellum volume		Uncinate fasciculus volume	
Peripapillary retinal nerve fiber layer thickness, per SD lower	Crude	3425	**−0.13 (−0.16 to −0.10)**	**<0.001**	**−0.16 (−0.19 to −0.12)**	**<0.001**	**−0.03 (−0.07 to 0.00)**	**0.050**	**−0.07 (−0.11 to −0.04)**	**<0.001**	**−0.07 (−0.11 to −0.04)**	**<0.001**	**−0.07 (−0.11 to −0.04)**	**<0.001**
	1	3425	**−0.14 (−0.17 to −0.11)**	**<0.001**	**−0.18 (−0.21 to −0.15)**	**<0.001**	**−0.08 (−0.11 to −0.05)**	**<0.001**	**−0.08 (−0.11 to −0.04)**	**<0.001**	**−0.10 (−0.13 to −0.07)**	**<0.001**	**−0.10 (−0.14 to −0.07)**	**<0.001**
	2	3425	**−0.14 (−0.17 to −0.11)**	**<0.001**	**−0.17 (−0.20 to −0.14)**	**<0.001**	**−0.08 (−0.11 to −0.05)**	**<0.001**	**−0.07 (−0.11 to −0.04)**	**<0.001**	**−0.10 (−0.13 to −0.07)**	**<0.001**	**−0.10 (−0.14 to −0.07)**	**<0.001**
Macular retinal nerve fiber layer thickness, per SD lower	Crude	3295	−0.01 (−0.05 to 0.02)	0.51	−0.01 (−0.04 to 0.03)	0.72	−0.02 (−0.06 to 0.01)	0.23	0.00 (−0.03 to 0.04)	0.92	**−0.04 (−0.07 to −0.001)**	**0.033**	−0.01 (−0.05 to 0.02)	0.48
	1	3295	−0.02 (−0.05 to 0.01)	0.23	−0.01 (−0.04 to 0.02)	0.44	0.00 (−0.03 to 0.03)	0.87	−0.01 (−0.04 to 0.02)	0.52	−0.03 (−0.06 to 0.00)	0.081	−0.01 (−0.04 to 0.02)	0.56
	2	3295	−0.02 (−0.05 to 0.01)	0.21	−0.01 (−0.04 to 0.02)	0.44	0.00 (−0.03 to 0.03)	0.90	−0.01 (−0.04 to 0.02)	0.51	−0.02 (−0.05 to 0.01)	0.10	−0.01 (−0.04 to 0.02)	0.60
Macular ganglion cell layer thickness, per SD lower	Crude	3295	**−0.18 (−0.22 to −0.15)**	**<0.001**	**−0.19 (−0.23 to −0.16)**	**<0.001**	**−0.09 (−0.12 to −0.05)**	**<0.001**	**−0.15 (−0.18 to −0.11)**	**<0.001**	**−0.15 (−0.18 to −0.11)**	**<0.001**	**−0.09 (−0.13 to −0.06)**	**<0.001**
	1	3295	**−0.10 (−0.13 to −0.07)**	**<0.001**	**−0.09 (−0.12 to −0.06)**	**<0.001**	**−0.05 (−0.08 to −0.01)**	**0.004**	**−0.09 (−0.12 to −0.06)**	**<0.001**	**−0.07 (−0.10 to −0.04)**	**<0.001**	**−0.05 (−0.08 to −0.01)**	**0.008**
	2	3295	**−0.09 (−0.13 to −0.06)**	**<0.001**	**−0.09 (−0.12 to −0.06)**	**<0.001**	**−0.05 (−0.08 to −0.01)**	**0.004**	**−0.09 (−0.12 to −0.05)**	**<0.001**	**−0.07 (−0.09 to −0.04)**	**<0.001**	**−0.05 (−0.08 to −0.01)**	**0.008**
Macular inner plexiform layer thickness, per SD lower	Crude	3295	**−0.19 (−0.23 to −0.16)**	**<0.001**	**−0.20 (−0.23 to −0.17)**	**<0.001**	**−0.11 (−0.14 to −0.07)**	**<0.001**	**−0.14 (−0.18 to −0.11)**	**<0.001**	**−0.17 (−0.20 to −0.13)**	**<0.001**	**−0.10 (−0.13 to −0.07)**	**<0.001**
	1	3295	**−0.10 (−0.13 to −0.06)**	**<0.001**	**−0.09 (−0.12 to −0.06)**	**<0.001**	**−0.05 (−0.08 to −0.02)**	**0.002**	**−0.08 (−0.12 to −0.05)**	**<0.001**	**−0.07 (−0.10 to −0.04)**	**<0.001**	**−0.05 (−0.08 to −0.01)**	**0.007**
	2	3295	**−0.09 (−0.12 to −0.06)**	**<0.001**	**−0.08 (−0.11 to −0.05)**	**<0.001**	**−0.05 (−0.08 to −0.02)**	**0.002**	**−0.08 (−0.11 to −0.04)**	**<0.001**	**−0.07 (−0.10 to −0.04)**	**<0.001**	**−0.05 (−0.08 to −0.01)**	**0.008**

*Notes*: Table 5 shows the associations of retinal thickness indices with brain metrics of regions implicated in the pathobiology of mild cognitive impairment and Alzheimer's disease (ie, hippocampal volume, thalamus volume, corpus callosum volume, cerebellum volume, and uncinate fasciculus volume). Values per SD of retinal thickness indices are shown in the legend of Table 2 in the main manuscript. Values per SD of brain index are shown in Table S1. Variables entered in models: Crude: none; Model 1: + age, sex, glucose metabolism status, educational level, spherical equivalent, MRI lag time; Model 2: model 1 + office systolic blood pressure, history of cardiovascular disease, waist circumference, smoking status, alcohol consumption, Total/HDL cholesterol ratio, lipid‐modifying medication, and antihypertensive medication. Bold denotes *P* < 0.05.

Abbreviations: CI, confidence interval; HDL, high‐density lipid; MRI, magnetic resonance imaging; N, population sample size; SD, standard deviation.

## 4 DISCUSSION

The present population‐based study has four main findings. First, lower pRNFL thickness was significantly associated with lower total white matter volume, whole brain node degree, global efficiency, and hippocampal volume. Second, lower mGCL and mIPL thicknesses were significantly associated with lower global cognitive performance, total grey and white matter volume, whole brain node degree, and hippocampal volume. Third, lower pRNFL, mGCL, and mIPL thicknesses were associated with higher clustering coefficient and local efficiency. Fourth, lower mRNFL thickness was not significantly associated with cognitive performance, total grey matter volume, total white matter volume, global or local structural connectivity indices, and hippocampal volume.

Our data are consistent with data from previous large population‐based studies on the associations of inner retinal layer thicknesses with cognitive performance^6^, ^8^, ^9^, ^10^, ^11^, ^12^, ^13^, ^14^ and total grey and white matter brain volume,^15^, ^16^, ^17^ and with data from a smaller case‐control study that evaluated the associations of retinal layer thicknesses with global white matter network indices.^31^ Importantly, the present study is the first to show the associations of the thicknesses of inner retinal layers with white matter network structure, as quantified from global and local structural connectivity indices.^4^

Biologically, lower thicknesses of inner retinal layers are thought to reflect lower numbers of retinal ganglion cells, which serve to transmit visual information (that is perceived by the photoreceptors and filtered in other retinal layers) to the lateral geniculate nucleus of the thalamus, from where visual information is transmitted to the visual cortex.^5^ Lower RNFL thickness represents lower numbers of retinal ganglion cell axons,^32^, ^33^ lower GCL thickness represents lower numbers of retinal ganglion cell bodies,^33^, ^34^ and lower IPL thickness represents lower numbers of synapses between retinal ganglion cell dendrites and bipolar cell axons.^33^, ^35^

Multiple pathobiological mechanisms are thought to contribute to neurodegeneration in the retina and the brain.^5^ First, inflammation, in part induced by systemic risk factors such as hyperglycemia,^36^ is thought to be detrimental for the neurovascular coupling unit in the blood‐brain barrier and in the blood‐retina barrier, predisposing to neuronal ischemia and, subsequently, neurodegeneration.^1^, ^37^ Second, in the retina and in the brain, accumulation of amyloid beta and tau plaques can lead to neurodegeneration and microvascular dysfunction, and both of these processes can result in dysfunction of the neurovascular coupling unit and predispose to (progression of) neurodegeneration.^1^, ^37^ Of note, as postulated in the two‐hit hypothesis of Alzheimer's disease, damage to the blood‐brain barrier or blood‐retina barrier may be the initial event, and the accumulation of amyloid beta and tau may be a secondary event.^1^ Third, neurodegeneration in brain regions to which the retina projects, such as the lateral geniculate nucleus of the thalamus, may lead to loss of axonal structures in the retina.^5^ In support of this concept, experimental data from monkeys and observational data from humans show that damage of brain regions that are part of the visual system was associated with a loss of neurons in the optic nerve head.^38^, ^39^ Fourth, RNFL thinning may be secondary to GCL thinning.^40^ Deterioration of retinal ganglion cell soma (ie, GCL thinning) may lead to impaired regulation of the intraneuronal milieu in the axon, which can lead to axonal degeneration (and ultimately can result in the loss of axons, which can be detected as lower RNFL thickness).^41^

Lower thicknesses of inner retinal layers were associated with a higher clustering coefficient and a higher local efficiency, possibly because these two locally determined network measures reflect compensatory remodeling of local brain networks in response to the loss of long‐range brain connections (ie, loss of global efficiency).^4^ Remodeling of local brain networks likely serves to maintain sufficiently high levels of information transmission within the brain in order to prevent a decline in cognitive performance.^4^ Such remodeling may consist of an increase in short‐range brain connections.^4^ Indeed, this interpretation is consistent with our previous findings that higher local efficiency was associated with worse cognitive performance and the presence of white matter hyperintensities.^24^ Of note, however, these results may seem contradictory as intuitively the deterioration of cerebral networks may be thought to result in fewer short‐range connections.^42^

Both mRNFL and pRNFL are measures of RNFL thickness; however, we found less strong associations of mRNFL with brain outcomes. A possible explanation may be that the assessment of mRNFL thickness may be more susceptible to measurement error because the mRNFL is considerably thinner parafoveally than peripapillarily (where pRNFL thickness is measured).^5^ Measurement error can lead to null findings via regression dilution bias.^43^ Indeed, consistent with this concept, the value of one SD of mRNFL thickness (an index of the accuracy of the assessment of RNFL thickness) was proportionately greater (relative to the mean value of RNFL thickness) than one SD of pRNFL thickness (for mRNFL: 1 SD = 4.23 micrometer, mean value 22.53, ratio: 18%; for pRNFL: 1 SD = 10.89 micrometer, mean 94.92; ratio: 9%).

Our findings support the concept that inner retinal layer thicknesses may be potential imaging biomarkers for monitoring subclinical neurodegenerative changes of the brain which have already occurred prior to the onset of clinical dementia.^5^ Use of inner retinal layer measurements may be a feasible monitoring tool as it is non‐invasive, relatively inexpensive, and easier to perform than other tests of early neuronal dysfunction such as MRI.^5^ Indeed, lower thicknesses of inner retinal layers were notably associated with brain metrics in regions implicated in the pathobiology of mild cognitive impairment and Alzheimer's disease. In particular, retinal thicknesses were also associated with hippocampal volume, which is an important clinical measure that can be used to assess neurodegeneration as part of the amyloid beta, tau, and neurodegeneration (ATN) classification framework proposed by the National Institute on Aging and Alzheimer's Association (NIA‐AA).^44^ Also in support of this concept, we additionally found that retinal neurodegeneration and cerebral neurodegeneration have shared risk factors.^45^, ^46^ We recently showed in The Maastricht Study, using data from up to ∼5600 individuals, that most risk factors for dementia, including hyperglycemia and hypertension, were associated with retinal neurodegeneration, as quantified from lower RNFL thickness and worse retinal sensitivity.^45^, ^46^ Of note, however, as we found relatively weak associations of retinal neuronal variables with brain variables, retinal neuronal imaging biomarkers may by themselves (as singular biomarkers) not be sufficiently informative to use in a clinical care setting. Future studies should aim to evaluate the added value of retinal imaging biomarkers on top of (a combination of) other non‐invasive and scalable biomarkers for dementia (such as blood‐based biomarkers for amyloid beta and tau in Alzheimer's disease).^47^ Further, our data indicate that future researchers may choose to focus on pRNFL, mGCL, and mIPL as biomarkers of early retinal neurodegeneration. For reasons of precision, mRNFL may be a less suitable biomarker for monitoring RNFL thickness than pRNFL thickness.

Strengths of this study are as follows: (1) the large size of this population‐based cohort with oversampling of individuals with type 2 diabetes; (2) the extensive number of potential confounders that were considered; and (3) the use of state‐of‐the‐art and novel methods to assess all variables included in this study (eg, the comprehensive assessment of brain MRI measures, including measures of white matter network structure).^48^

The study has certain limitations. First, due to the cross‐sectional nature of the study, causal inferences should be made with caution.^49^ Second, we may have underestimated the strength of the associations if such associations were similar or stronger in participants that were excluded from the study population (who were not substantially less healthy, but generally had a somewhat worse cardiovascular risk profile).^48^ The associations with MRI measures are the most susceptible to this form of selection bias, as certain individuals who may be less healthy (eg, those with a pacemaker) did not undergo MRI imaging.^48^ Third, although we took an extensive set of confounders into account, we cannot fully exclude bias due to unmeasured confounding (eg, environmental factors such as air pollution).^50^ Last, we studied Caucasian individuals aged 40 to 75 years. Therefore, the generalizability of our results to other populations requires further study.

In summary, the present population‐based study demonstrated that retinal neurodegeneration, estimated from lower thicknesses of inner retinal layers, was associated with worse cognitive performance, lower total grey and white matter brain volume, and altered brain white matter network structure. These results are consistent with the concept that the retina may provide non‐invasive and scalable biomarkers that are informative of cerebral neurodegenerative changes in the pathobiology of dementia.

## AUTHOR CONTRIBUTIONS

F.C.T. vd.H., I.L.M. Steens, and C.D.A.S. contributed to conception and design, participated in acquisition of data, analyzed and interpreted data, drafted the manuscript, revised the manuscript critically for important intellectual content, and provided final approval of the version to be published. F.C.T. vd.H also is the guarantor of this work and, as such, had full access to all the data in the study and takes responsibility for the integrity of the data and the accuracy of the data analysis. B.L., M.P.J.v.B., M.T.S., S.K., A.A.K., C.J.H.vd.K., T.v.S., P.C.D., M.C.J.M.v.D., S.J.P.M.E., T.J.M.B., C.A.B.W., M.M.J.v.G., A.K., J.F.A.J., and W.H.B. contributed to conception and design, revised the manuscript critically for important intellectual content, and provided final approval of the version to be published.

## CONFLICT OF INTEREST STATEMENT

The authors declare no conflicts of interest. Author disclosures are available in the Supporting information.

## CONSENT STATEMENT

All human subjects provided informed consent.

## Supporting information

Supplementary Information

Supplementary Information
