# Supplementary material for: Thinner inner retinal layers are associated with lower cognitive performance, lower brain volume, and altered white matter network structure—The Maastricht Study
Source: Alzheimers Dement. 2023 Aug 23;20(1):316–29. doi: 10.1002/alz.13442 (PMC10917009; doi:10.1002/alz.13442)
Supplement: Supplementary file 1 — Supplementary Information [file ALZ-20-316-s002.docx]

**Supplemental Material**

Contents

- Supplemental Methods
- Supplemental Results
- Supplemental Figures
  - Supplemental Figure S1. Examples of good and poor quality peripapillary OCT scans
  - Supplemental Figure S2 Examples of peripapillary OCT scans of poor quality or with scan errors
  - Supplemental Figure S3 Examples of macular OCT scans of poor quality or with scan errors
  - Supplemental Figure S4 Non-linear associations of pRNFL and mRNFL with cognitive performance
- Supplemental Tables
  - Supplemental Table S1 Additional general study population characteristics according to tertiles of pRNFL thickness in the study population with complete data on cognitive performance
  - Supplemental Table S2 Retinal and brain indices according to mild cognitive impairment status in the study population with complete data on pRNFL thickness and MRI data
  - Supplemental Table S3 General study population characteristics of the included and excluded participants for the study population with complete data on pRNFL, potential confounders, and cognitive performance
  - Supplemental Table S4 General study population characteristics of the included and excluded participants for the study population with complete data on pRNFL, potential confounders, and MRI measures
  - Supplemental Table S5 P-values for interaction by sex, prediabetes, and type 2 diabetes in the associations of retinal thickness indices with cognitive performance and brain MRI measures
  - Supplemental Table S6 Associations of retinal thickness indices with memory, executive function, and information processing speed
  - Supplemental Table S7 Associations of retinal thickness indices with global cognitive performance, with additional adjustment for diet and physical activity (model 3A); the presence of a major depressive episode (model 3B); biomarkers of inflammation (model 3C); ocular pathology (diabetic retinopathy, glaucoma, age-related macular degeneration; model 3D); kidney variables (i.e. eGFR, albuminuria; model 3E); or after exclusion of individuals with ocular pathology (i.e. diabetic retinopathy, glaucoma, and/or age-related macular degeneration; model 3F)
  - Supplemental Table S8 Associations of retinal thickness indices with MRI measures, with additional adjustment for diet and physical activity (model 3A); biomarkers of inflammation (model 3B); ocular pathology (diabetic retinopathy, glaucoma, age-related macular degeneration; 3C); kidney variables (i.e. eGFR, albuminuria; model 3D); or after exclusion of individuals with ocular pathology (i.e. diabetic retinopathy, glaucoma, and/or age-related macular degeneration; model 3E)

.

**Supplemental Methods**

Assessment of retinal thickness indices

All participants were asked to refrain from smoking and drinking caffeine-containing beverages three hours before the measurement. A light meal (breakfast or lunch), low in fat content, was allowed if taken at least 90 minutes prior to the start of the measurements. Pupils were dilated with topical 0.5% tropicamide and 2.5% phenylephrine 15 minutes prior to the start of the examination.

We assessed retinal thickness indices with optical coherence tomography (OCT; Spectralis unit and Eye Explorer version 5.7.5.0 software; Heidelberg Engineering, Heidelberg, Germany). Measurements were performed by trained research assistants according to a standard operating procedure.

*Peripapillary RNFL thickness*

The peripapillary retinal nerve fiber layer thickness (pRNFL; μm) in both eyes was measured within a 3.45 mm diameter circular scan (12°, 768 voxels, 100 automatic real-time tracking) centered on the optic nerve head. The peripapillary OCT scans were reviewed and scored for the presence of measurement errors by experienced graders based on a predefined protocol. Graders were masked to clinical information of the participants. OCT images were excluded if one of the following criteria was present: scan errors (i.e. incomplete scan, poor centering of the circular scan on the optic nerve head, retinal layer incorrectly defined, or technical problem with the OCT device) and/or poor imaging quality (signal-to-noise ratio<15 dB; examples are shown in Supplemental Figures S1 and S2). The interrater reliability for the assessment of peripapillary retinal nerve fiber layer thickness and macular retinal thickness were 0.96 and 0.98 respectively.^1^

*Macular retinal layer thickness*

We assessed the average thickness of the retina of both eyes in the central macular area (Early Treatment Diabetic Retinopathy Study [ETDRS] sectors 1-5) using a fovea-centered macular volume scan (73 sections, 60 μm). We assessed the thickness of the following macular retinal layers: macular RNFL thickness (mRNFL); ganglion cell layer (mGCL); inner plexiform layer (mIPL). We used the average thickness of both eyes to calculate the thickness of retinal layers, however, if data were only available for one eye, we used data from this eye only.

We determined the thickness of individual retinal layers as follows. First, we exported data on individual voxels from the OCT device using custom software from Heidelberg Engineering. Second, we in MATLAB (MATLAB and Statistics Toolbox Release 2012b, The MathWorks, Inc., Natick, USA) calculated the thickness of individual retinal layers per voxel. Third, we exported data on the thickness of individual retinal layers for voxels located in sectors 1-5 of the ETDRS grid and per eye summarized these data into mean thickness in sectors 1-5 of the ETDRS grid for all individual retinal layers. With the presently used software the thickness of individual retinal layers could only be determined if at minimum 72 out of 73 slices were correctly outlined.

To check whether a more strict selection of macular OCT images, would impact our results we graded a subset of macular OCT scans (from ~2,500 participants) for the presence of measurement errors, as previously described.^2^ Measurement errors were defined as: incomplete scan (i.e. <73 slices); incorrect centering on the fovea; poor outlining of the retina; technical problem of the OCT device visible; poor image quality (i.e. signal-to-noise ratio [dB]<15). Exemplary images are shown in Supplemental Figure S3. Overall >95% of retinal scans were of sufficient quality and exclusion of scans of insufficient quality did not affect the results (data not shown). Hence, we did not exclude any images for the main analyses.

Assessment of global cognitive performance

We assessed three domains of cognitive performance, i.e. memory, information processing speed, and executive function with a concise neuropsychological test battery.^3^ We evaluated memory with the Verbal Learning Test;^4^ information processing speed with parts I and II of the Stroop Color-Word Test,^5^ parts A and B of the Concept Shifting Test,^6^ and the Letter-Digit Substitution Test;^7^ and executive function with part III of the Stroop Color-Word Test and part C of the Concept Shifting Test.

For statistical efficiency, we used the results of these tests to construct a composite score for global cognitive performance. We constructed a composite score for global cognitive performance to reduce noise (i.e. measurement error) and because we assume that the individual measures used in tests to assess global cognitive performance represent similar underlying constructs.^8, 9^ We constructed a composite in four steps. First, we expressed raw test results in standard deviations (z-score). Second, we inverted z-scores of the Stroop Color-Word Test and Concept Shifting Test so that higher scores indicate better cognitive performance. Third, we calculated domain-specific cognitive performance composite z-scores by averaging z-scores of (sub)tests within that domain. Last, we computed global cognitive performance by averaging z-scores for memory, processing speed, and executive function.

Assessment of MRI measures

Participants were eligible for magnetic resonance imaging (MRI) if none of the following contra-indications was present: a cardiac pacemaker or implantable cardioverter-defibrillator, a neurostimulator, a non-detachable insulin pump, metallic vascular clips or stents in the head, a cochlear implant, a metal-containing intra-uterine device, metal splinters or shrapnel, dentures with magnetic clip, an inside bracket, pregnancy, epilepsy, or claustrophobia.

Magnetic resonance images were made with a 3-T scanner (Magnetom Prismafit Syngo MR D13D; Siemens Healthcare, Erlangen, Germany) using a 64-element head and neck coil. Brain structure was assessed with T1-weighted images (TR/TE/TI2300/2.98/900 ms, 1.00 mm cubic voxel, 176 continuous slices, matrix size of 240 × 250 and reconstructed matrix size of 512 × 512). Structural connectivity was assessed with diffusion-weighted magnetic resonance imaging (MRI), which consists of a diffusion-sensitized echo-planar imaging sequence (repetition time msec/echo time msec, 6100/57; 65 slices; 100 × 100 matrix; 2.00-mm voxel size; 64 diffusion sensitizing gradient directions [b = 1200 sec/mm2]) using three images with a b value of 0.

*Total grey and white matter volume*

We estimated total grey and white matter volume relative to intracranial volume from T1 images using the FreeSurfer software package (Martinos Center for Biomedical Imaging, Boston, USA; 1 voxel = 1.00 mm^3^ = 0.001 ml).^10^ Intracranial volume was calculated as the sum of grey matter, white matter (including white matter hyperintensity volume), and cerebrospinal fluid volumes. The segmentations were visually inspected. Brain segmentation was performed with FreeSurfer v6.0 (Fischl, 2012) using T1w and FLAIR images as input. The optional arguments “‑FLAIRpial” and “‑3T” were used to optimize segmentation quality. Brain segmentations with insufficient quality, i.e. Euler numbers below 1.5 quartile (-80 for left hemisphere and -68 for right hemisphere) were excluded.^11^

*Quantification of brain regions implicated in the pathobiology of Alzheimer’s disease and mild cognitive impairment*

We calculated the volume or area of brain regions implicated in the pathobiology of Alzheimer’s disease and mild cognitive impairment, regions which were all selected from a recent systematic review,^12^ as follows. To start, we calculated hippocampal volume, thalamus volume, and uncinate fasciculus volume by summing the volumes of these structures in the left and right hemisphere. Next, as data on the volumes of individual cerebellar lobules I,II,III,IV, and VI were not presently available, we calculated total cerebellum lobule volume. To calculate total cerebellum lobule volume, we summed the volume of the cerebellar cortex of both hemispheres and the volume of the cerebellar white matter of both hemispheres. Then, we calculated the total cingulate cortex surface area as the sum of the surface area of the isthmus of both hemispheres and the surface area of the posterior, rostral anterior, and caudal anterior regions of the cingulate cortices of both hemispheres. Last, we calculated corpus callosum volume by summing the volumes of the posterior, mid-posterior, central, mid-anterior, and anterior regions of the corpus callosum.

*Structural connectivity*

Network analysis was performed using the Brain Connectivity Toolbox (version 2017-15-01) in MATLAB (Release 2016a, The MathWorks, Inc., Natick, Massachusetts, USA).^13^ In this method, the brain was represented as a graph, which is a network of nodes (i.e. gray matter brain regions) connected by edges (i.e. white matter connections between brain regions).^14^ The following graph theoretical measures were calculated: 1) the whole brain node degree, which is a measure for the average number of edges connected to a node (i.e. in a network with a high whole brain node degree, brain regions are connected to many other brain regions in the network); 2) global efficiency, a measure of the average inverse shortest path length, inversely related to the path length; 3) the clustering coefficient, which is a measure of local network connectivity (i.e. a network with a high clustering coefficient is characterized by densely connected local clusters); and 4) local efficiency, which is a measure of the mean global efficiency of subgraphs computed on the immediate neighbors of a node and is related to the cluster coefficient.^14^ Last, for each participant, the graph measures were normalized to comparable values from randomly generated networks (N = 100) to evaluate whether the network had small-world properties.

**Assessment of covariates**

As described previously,^3^ we assessed educational level (low, intermediate, high), socio-economic status (income level and occupational status [low, intermediate, high]),^15^ smoking status (never, former, current), alcohol consumption (none, low, high), history of cardiovascular disease (yes/no), and the presence of age-related macular degeneration (yes/no) by questionnaire; assessed dietary habits (“dietary intake”) with the Dutch Healthy Diet index sum score, a measure of adherence to the Dutch dietary guidelines 2015,^16^ based on a validated food frequency questionnaire;^17^ assessed glucose metabolism status (normal glucose metabolism status; prediabetes; type 2 diabetes; and other types of diabetes than type 2) using data on fasting plasma glucose and 2-hour post load glucose; assessed the presence of a major depressive disorder with the Mini-International Neuropsychiatric Interview ^18^; assessed lipid-modifying medication use (yes/no), antihypertensive medication use (yes/no), and intraocular pressure-lowering medication use (yes/no) as part of a medication interview; assessed weight, height, and waist circumference during a physical examination; calculated body mass index (BMI; in kg/m^2^) based on body weight and height; measured office and 24-hour ambulatory blood pressure (in mm Hg); measured total daily physical activity (hours/day) with an accelerometer;^19^ measured lipid profile (i.e. total cholesterol/ high density lipid [HDL] ratio) and plasma biomarkers of low-grade inflammation(i.e., high-sensitive C-reactive protein, serum amyloid A, interleukin-6, interleukin-8 and tumor necrosis factor alpha [presently available for n= 3,451 participants]), ^20^ fasting plasma glucose, 2-hour post load glucose, and glycated hemoglobin (HbA1c) in fasting venous blood samples; measured urinary albumin excretion in two 24-hour urine collections; calculated the estimated glomerular filtration rate (eGFR) based on serum creatinine only as data on cystatin C were presently not available; ^21^ assessed the presence of retinopathy in both eyes from fundus images; and assessed spherical equivalent and intraocular pressure in both eyes using an automated refractor and noncontact tonometer (Tonoref II; Nidek, Gamagordi, Japan). Glaucoma was defined as use of intraocular pressure-lowering medication, intraocular pressure higher than 21 mmHg in any eye, or both (96% of all participants had data on intraocular pressure available for both eyes). Spherical equivalent was defined as the mean spherical equivalent of both eyes or as the spherical equivalent of the eye for which data were available (99% of all participants had data on spherical equivalent available for both eyes).

Statistical analyses

We checked assumptions for linear regression analyses and used collinearity diagnostics (i.e., tolerance <0.10 and/or variance inflation factor >10) to detect for excessive multicollinearity between covariates. Last, we used complete case analysis.

Additional analyses

We performed a range of additional analyses. First, we analyzed the associations of macular retinal thickness and pRNFL thickness with indices of regional brain structures, i.e. hippocampal volume, thalamus volume, cingulate cortex surface area, corpus callosum volume, cerebellum volume, and uncinate fasciculus volume. Second, we analyzed the associations of retinal thickness indices with individual cognitive domains, i.e. memory, executive function, and information processing speed. Third, we repeated the analyses with additional adjustment for physical activity and diet score. Adjustment for these potential confounders was not included in the main analysis, because data were missing in a relatively large number of participants (up to n=856 missed data on one or more of these variables). Fourth, we analyzed the associations of retinal thickness indices with cognitive performance after adjustment for the presence of a major depressive disorder. The presence of a major depressive disorder may be a confounder and/or (in part) a potential mediator.^22^ Fifth, we additionally adjusted for inflammation markers, retinal pathology (i.e. glaucoma, diabetic retinopathy, and age-related macular degeneration), and kidney variables (estimated glomerular filtration rate, albuminuria) as they may be confounders and/or (in part) potential mediators.^22^ Sixth, we performed additional analyses in which we excluded individuals with retinal pathology (glaucoma, diabetic retinopathy, and/or age-related macular degeneration). Last, we performed analyses in which we replaced waist circumference by body-mass index (BMI); glucose metabolism status by continuous measures of glycaemia (i.e., fasting plasma glucose, 2-h post load glucose, or HbA1c); office systolic blood pressure by office diastolic blood pressure, 24-hour ambulatory systolic or diastolic blood pressure; and educational status by occupational status or income level.

**References**

1. Hong JT, Sung KR, Cho JW, Yun SC, Kang SY and Kook MS. Retinal nerve fiber layer measurement variability with spectral domain optical coherence tomography. *Korean J Ophthalmol*. 2012;26:32-8.

2. De Clerck EEB, Schouten J, Berendschot T, Goezinne F, Dagnelie PC, Schaper NC, Schram MT, Stehouwer CDA and Webers CAB. Macular thinning in prediabetes or type 2 diabetes without diabetic retinopathy: the Maastricht Study. *Acta Ophthalmol*. 2018;96:174-182.

3. Schram MT, Sep SJ, van der Kallen CJ, Dagnelie PC, Koster A, Schaper N, Henry RM and Stehouwer CD. The Maastricht Study: an extensive phenotyping study on determinants of type 2 diabetes, its complications and its comorbidities. *Eur J Epidemiol*. 2014;29:439-51.

4. Van der Elst W, van Boxtel MP, van Breukelen GJ and Jolles J. Rey's verbal learning test: normative data for 1855 healthy participants aged 24-81 years and the influence of age, sex, education, and mode of presentation. *J Int Neuropsychol Soc*. 2005;11:290-302.

5. Van der Elst W, Van Boxtel MP, Van Breukelen GJ and Jolles J. The Stroop color-word test: influence of age, sex, and education; and normative data for a large sample across the adult age range. *Assessment*. 2006;13:62-79.

6. Van der Elst W, Van Boxtel MP, Van Breukelen GJ and Jolles J. The Concept Shifting Test: adult normative data. *Psychol Assess*. 2006;18:424-32.

7. van der Elst W, van Boxtel MP, van Breukelen GJ and Jolles J. The Letter Digit Substitution Test: normative data for 1,858 healthy participants aged 24-81 from the Maastricht Aging Study (MAAS): influence of age, education, and sex. *J Clin Exp Neuropsychol*. 2006;28:998-1009.

8. Riordan HJ. Constructing composites to optimise cognitive outcomes. *J Clin Stud* 2017;9:40-45.

9. Hutcheon JA, Chiolero A and Hanley JA. Random measurement error and regression dilution bias. *BMJ*. 2010;340:c2289.

10. Vrooman HA, Cocosco CA, van der Lijn F, Stokking R, Ikram MA, Vernooij MW, Breteler MM and Niessen WJ. Multi-spectral brain tissue segmentation using automatically trained k-Nearest-Neighbor classification. *Neuroimage*. 2007;37:71-81.

11. Monereo-Sanchez J, de Jong JJA, Drenthen GS, Beran M, Backes WH, Stehouwer CDA, Schram MT, Linden DEJ and Jansen JFA. Quality control strategies for brain MRI segmentation and parcellation: Practical approaches and recommendations - insights from the Maastricht study. *Neuroimage*. 2021;237:118174.

12. Talwar P, Kushwaha S, Chaturvedi M and Mahajan V. Systematic Review of Different Neuroimaging Correlates in Mild Cognitive Impairment and Alzheimer's Disease. *Clin Neuroradiol*. 2021;31:953-967.

13. Rubinov M and Sporns O. Complex network measures of brain connectivity: uses and interpretations. *Neuroimage*. 2010;52:1059-69.

14. Farahani FV, Karwowski W and Lighthall NR. Application of Graph Theory for Identifying Connectivity Patterns in Human Brain Networks: A Systematic Review. *Front Neurosci*. 2019;13:585.

15. Qi Y, Koster A, van Boxtel M, Kohler S, Schram M, Schaper N, Stehouwer C and Bosma H. Adulthood Socioeconomic Position and Type 2 Diabetes Mellitus-A Comparison of Education, Occupation, Income, and Material Deprivation: The Maastricht Study. *Int J Environ Res Public Health*. 2019;16.

16. Looman M, Feskens EJ, de Rijk M, Meijboom S, Biesbroek S, Temme EH, de Vries J and Geelen A. Development and evaluation of the Dutch Healthy Diet index 2015. *Public Health Nutr*. 2017;20:2289-2299.

17. van Dongen MC, Wijckmans-Duysens NEG, den Biggelaar LJ, Ocke MC, Meijboom S, Brants HA, de Vries JH, Feskens EJ, Bueno-de-Mesquita HB, Geelen A, Stehouwer CD, Dagnelie PC and Eussen SJ. The Maastricht FFQ: Development and validation of a comprehensive food frequency questionnaire for the Maastricht study. *Nutrition*. 2019;62:39-46.

18. DVSheehan Y, KHSheehan P, JJanavs E and THergueta R. GCDunbar (1998). The Mini-International Neuropsychiatric Interview (MINI): the development and validation of a structured diagnostic psychiatric interview for DSM-IV and ICD-10. *J Clin Psychiatry*. 59:22-33.

19. van der Berg JD, Willems PJ, van der Velde JH, Savelberg HH, Schaper NC, Schram MT, Sep SJ, Dagnelie PC, Bosma H, Stehouwer CD and Koster A. Identifying waking time in 24-h accelerometry data in adults using an automated algorithm. *J Sports Sci*. 2016;34:1867-73.

20. van Dooren FE, Schram MT, Schalkwijk CG, Stehouwer CD, Henry RM, Dagnelie PC, Schaper NC, van der Kallen CJ, Koster A, Sep SJ, Denollet J, Verhey FR and Pouwer F. Associations of low grade inflammation and endothelial dysfunction with depression - The Maastricht Study. *Brain Behav Immun*. 2016;56:390-6.

21. Shahbaz H and Gupta M. Creatinine Clearance *StatPearls* Treasure Island (FL); 2021.

22. Schisterman EF, Cole SR and Platt RW. Overadjustment bias and unnecessary adjustment in epidemiologic studies. *Epidemiology*. 2009;20:488-95.

**Supplemental Results**

We generally had consistent findings in additional analyses (Supplemental Tables S6-S8). First, we found that lower thicknesses of most inner retinal layers (i.e. all layers except for mRNFL) were associated with lower brain volume or surface area of brain regions implicated in the pathobiology of Alzheimer’s disease and mild cognitive impairment (i.e. hippocampal volume, thalamus volume, cingulate cortex surface area, corpus callosum volume, cerebellum volume, and uncinate fasciculus volume; Table 5). Second, we had similar findings when we analyzed associations of thicknesses of retinal layers with individual cognitive domains as outcome instead of global cognitive performance (Supplemental Table S6). Third, we had numerically similar findings to those shown in the main analyses when we additionally adjusted for physical activity and diet; the presence of a major depressive disorder; biomarkers of inflammation; the presence of retinal disease (i.e. glaucoma, diabetic retinopathy, and/or age-related macular degeneration); and kidney variables (estimated glomerular filtration rate, albuminuria). Results of the latter analyses are shown in Supplemental Tables S7 and S8. Fifth, we performed additional analyses in which we excluded individuals with retinal pathology (glaucoma, diabetic retinopathy, and/or age-related macular degeneration; Supplemental Table S7 and S8). Last, we had numerically similar findings in analyses in which we replaced waist circumference by BMI; glucose metabolism status by continuous measures of glycaemia (i.e. fasting plasma glucose, 2-h post load glucose, or HbA1c); office systolic blood pressure by office diastolic blood pressure, 24-hour ambulatory systolic or diastolic blood pressure; and educational status by occupational status or income level (results of the latter analyses are not shown).

**Supplemental Figures and Table**

.

## Supplemental Figures

**
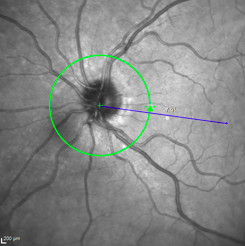
**
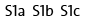

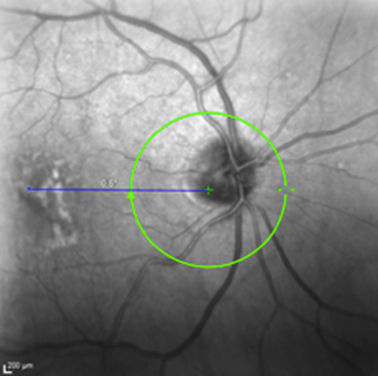

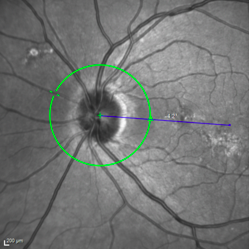


S1A

S1B

S1C

**Supplemental Figure S1. Examples of good and poor quality peripapillary OCT scans.** Supplemental Figure S1 shows examples of quality of centring of the circular scan on the optic nerve head: S1A shows good quality, S1B shows poor quality, and S1C shows very poor quality.


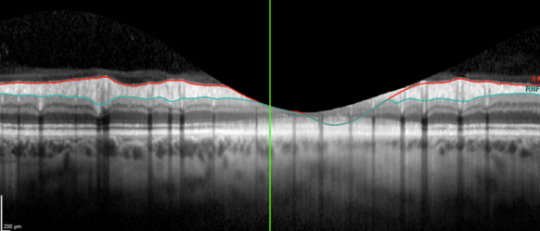

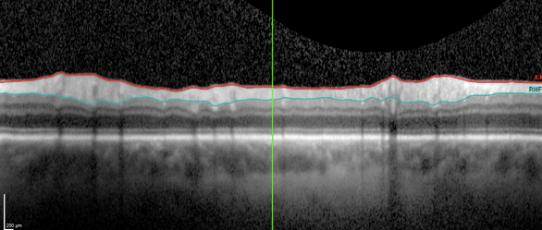


S2A

S2B


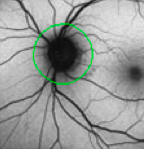


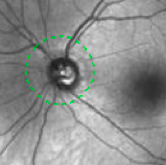

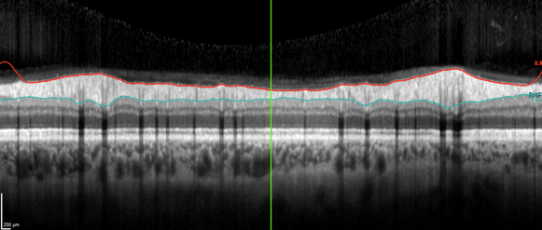


S2E

S2D

S2C


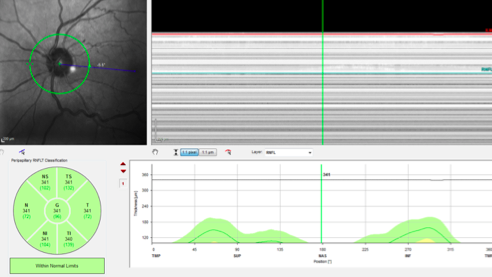

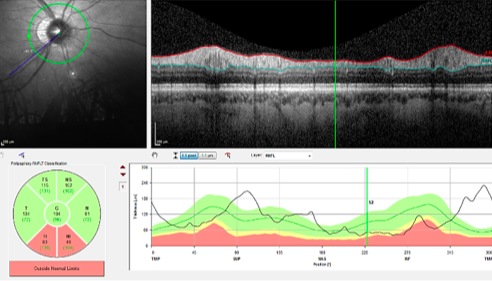


S2G

S2F

**Supplemental Figure S2 Examples of peripapillary OCT scans of poor quality or with scan errors.** S2A: Example of poor imaging quality (Signal-to-noise ratio<15 dB); S2B: OCT device too close to the eye; S2C: RNFL layer incorrectly defined; S2D: incorrect circle position (dashed line); S2E: autofluorescence on; S2F: participant does not look in the correct direction; S2G: technical problem with OCT device. OCT, optical coherence tomography; RNFL, retinal nerve fibre layer thickness.


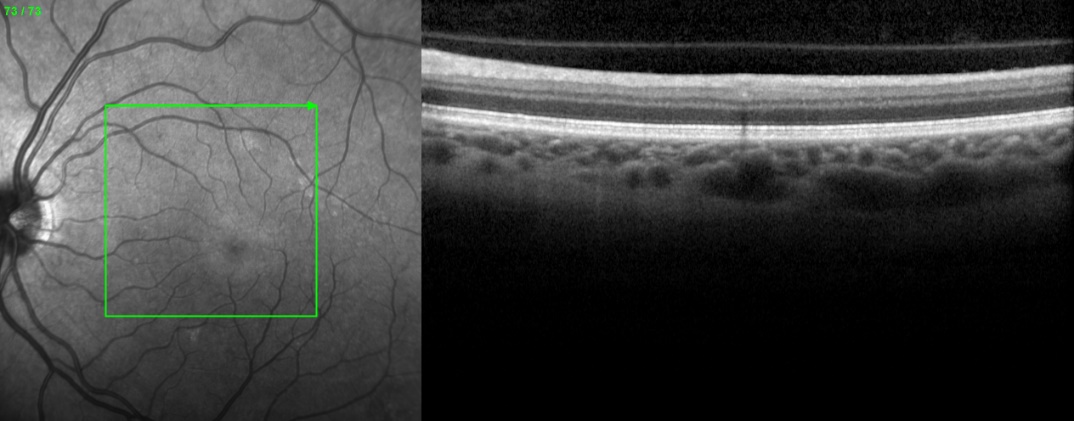

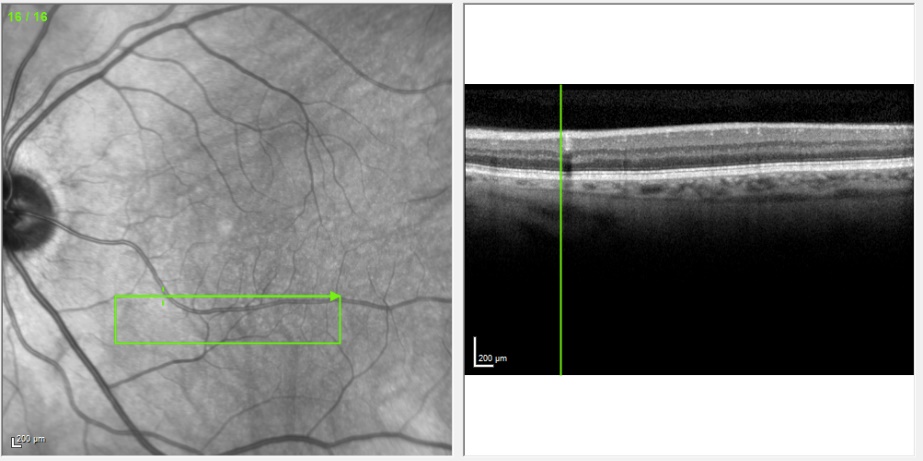

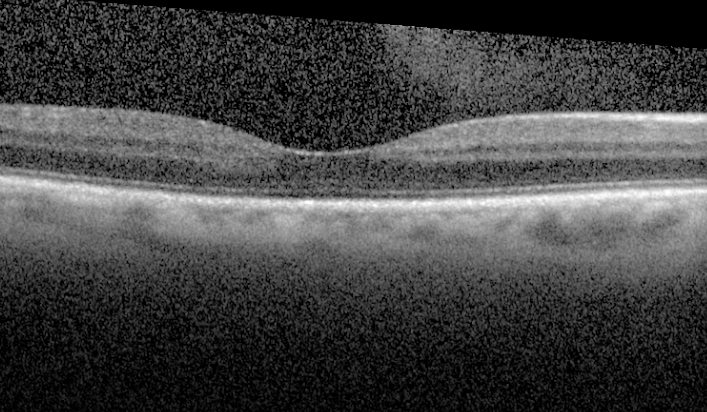


S3A

S3C

S3B


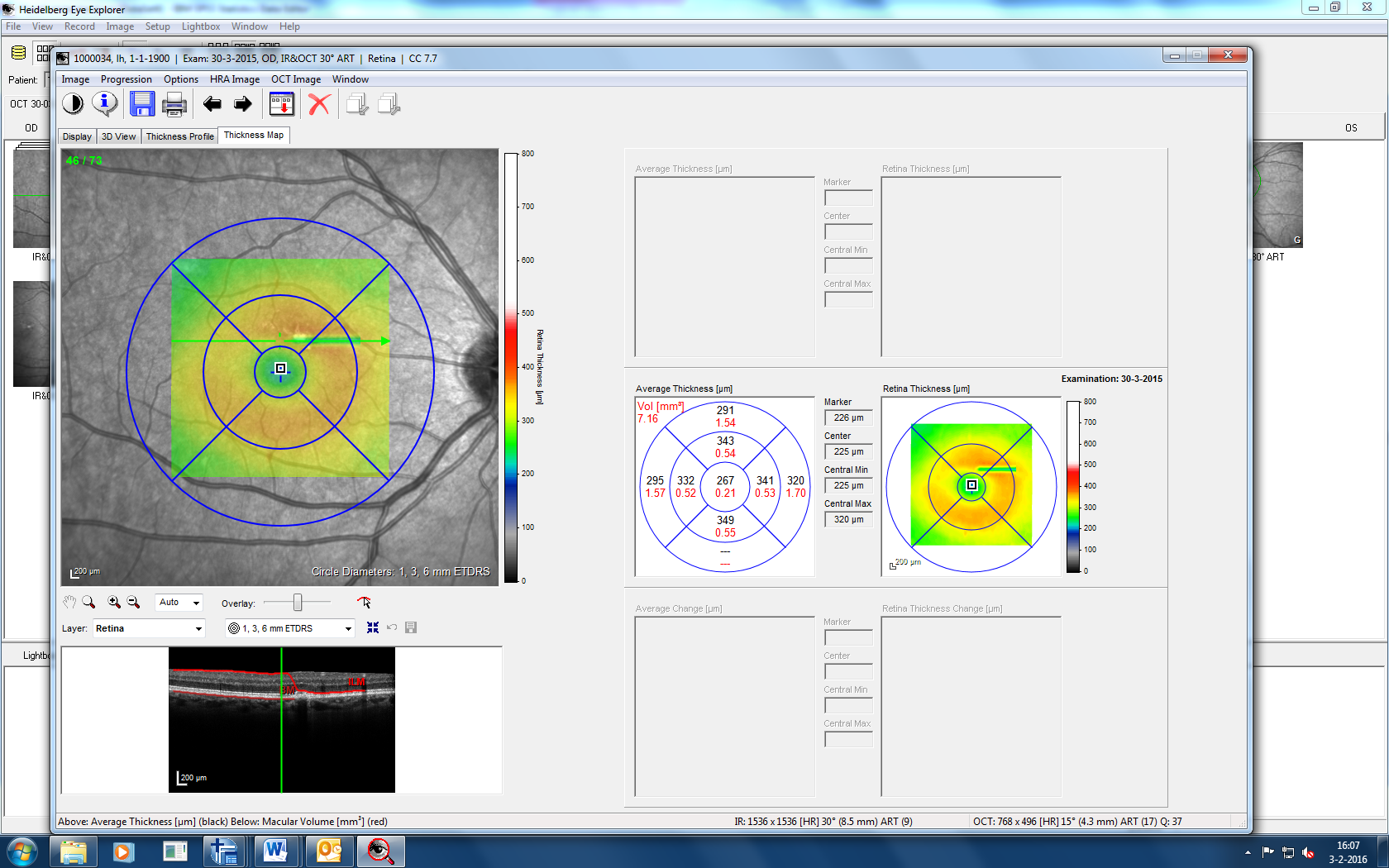

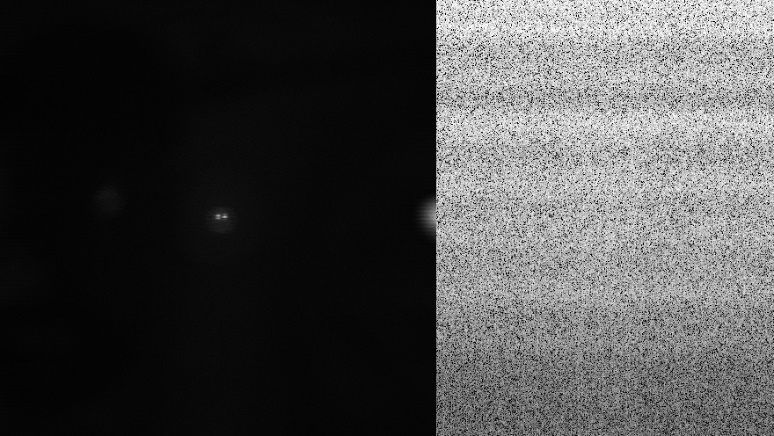


S3E

S3D

**Supplemental Figure S3 Examples of macular OCT scans of poor quality or with scan errors.** Figure S3A.poor image quality (signal-to-noise ratio<15 dB); Figure S3B. incomplete macular OCT scan; Figure S3C. incorrect position of the OCT scan on the retina (i.e. the scan is not fovea-centered); Figure S3D. yechnical problem with the OCT scan; Figure S3E. incorrect outlining of the retina.

Abbreviations*:* OCT, optical coherence tomography; ILM, internal limiting membrane.

**
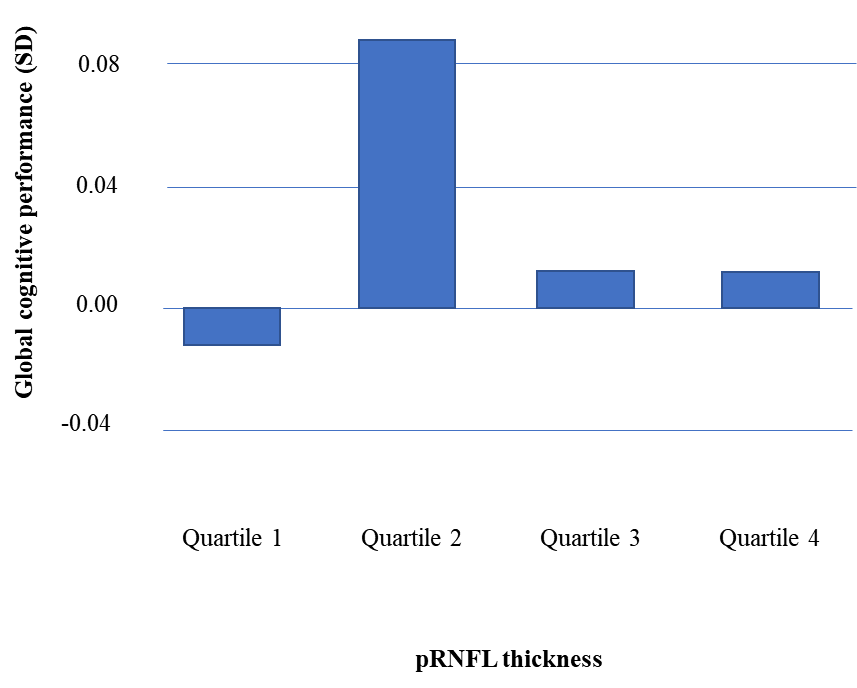

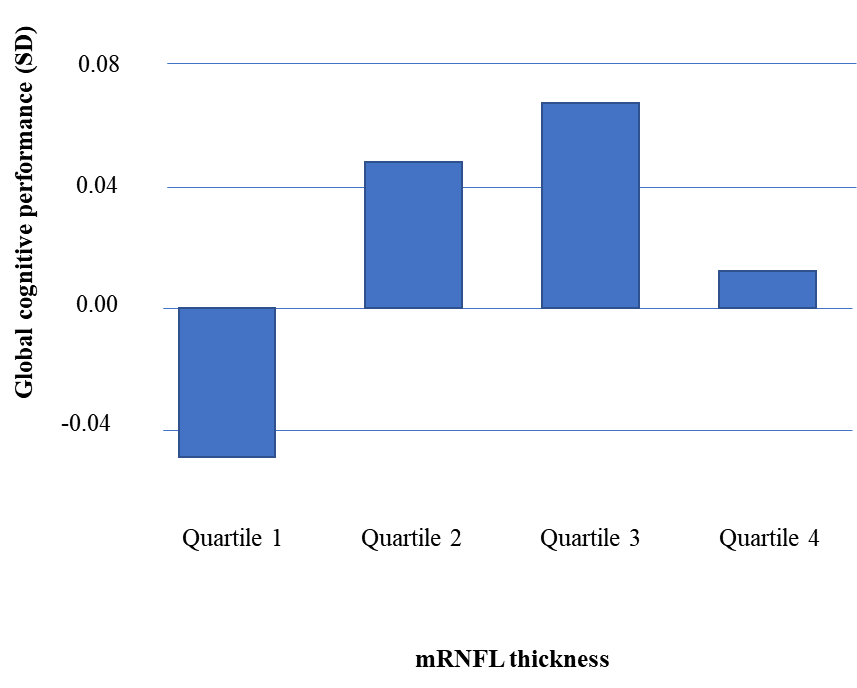
**

**Supplemental Figure S4.1 Supplemental Figure S4.2**

**Supplemental Figure S4 Non-linear associations of pRNFL and mRNFL with cognitive performance.** Supplemental Figure S4 shows the non-linear associations of pRNFL (S4.1)and mRNFL (S4.2) with global cognitive performance.

pRNFL and mRNFL were divided in quartiles.

Abbreviations: pRNFL, peripapillary retinal nerve fiber layer thickness; mRNFL, macular retinal nerve fiber layer thickness.

**Supplemental Table S1 Additional general study population characteristics according to tertiles of pRNFL thickness in the study population with complete data on cognitive performance**

|  | pRNFL thickness | | | |
| --- | --- | --- | --- | --- |
| Characteristic | Overall, N = 4,697 | Tertile 1, N=1,566 | Tertile 2, N=1,566 | Tertile 3, N=1,565 |
| BMI (kg/m2) | 26.77 ± 4.38 | 26.88 ± 4.37 | 26.61 ± 4.30 | 26.82 ± 4.45 |
| Office diastolic blood pressure (mmHg) | 75.58 ± 9.86 | 76.39 ± 9.92 | 75.15 ± 9.74 | 75.18 ± 9.87 |
| 24-h systolic blood pressure (mm Hg) | 119.09 ± 11.32 | 119.71 ± 11.24 | 119.00 ± 11.13 | 118.55 ± 11.57 |
| 24-h diastolic blood pressure (mm Hg) | 73.80 ± 7.09 | 74.11 ± 6.91 | 73.87 ± 7.17 | 73.41 ± 7.18 |
| Income (euros) | 2,029.75 ± 831.89 | 2,018.33 ± 846.09 | 2,056.75 ± 836.48 | 2,013.82 ± 811.93 |
| Occupational level |  |  |  |  |
| low | 469 (29) | 156 (27) | 152 (29) | 161 (33) |
| intermediate | 592 (37) | 202 (35) | 211 (40) | 179 (37) |
| high | 537 (34) | 226 (39) | 161 (31) | 150 (31) |
| fasting glucose (mmol/L) | 5.40 (5.00 – 6.10) | 5.40 (5.00 – 6.40) | 5.30 (4.90 – 6.00) | 5.30 (5.00 – 6.00) |
| 2-hour post load glucose (mmol/L) | 6.1 (4.9 – 8.4) | 6.3 (5.1 – 9.1) | 6.0 (4.9 – 8.1) | 6.0 (4.8 – 8.0) |
| HbA1c_mol | 38.94 ± 8.80 | 39.70 ± 9.42 | 38.54 ± 8.61 | 38.60 ± 8.31 |
| HbA1c_perc | 5.71 ± 0.81 | 5.78 ± 0.86 | 5.68 ± 0.79 | 5.68 ± 0.76 |
| Intraocular pressure | 14.06 ± 3.06 | 14.46 ± 3.11 | 13.91 ± 3.00 | 13.80 ± 3.04 |
| Diet score (DHD) | 84.11 ± 15.01 | 83.07 ± 14.84 | 84.82 ± 15.05 | 84.42 ± 15.09 |
| Physical activity (min/day) | 119.14 ± 40.50 | 117.59 ± 39.72 | 120.53 ± 40.11 | 119.29 ± 41.58 |
| C-reactive protein (µg/ml) | 1.17 (0.60 – 2.69) | 1.18 (0.66 – 2.76) | 1.14 (0.59 – 2.60) | 1.18 (0.56 – 2.63) |
| Human tumor necrosis factor alpha (pg/ml) | 2.17 (1.86 – 2.55) | 2.22 (1.92 – 2.59) | 2.14 (1.85 – 2.50) | 2.12 (1.83 – 2.53) |
| Human interleukin-6 (pg/ml) | 0.57 (0.38 – 0.88) | 0.61 (0.42 – 0.98) | 0.52 (0.35 – 0.80) | 0.56 (0.38 – 0.85) |
| Human interleukin-8 (pg/ml) | 4.00 (3.18 – 5.21) | 4.14 (3.24 – 5.35) | 3.94 (3.15 – 5.17) | 3.95 (3.16 – 4.99) |
| Serum amyloid A (µg/ml) | 3.2 (2.0 – 5.3) | 3.4 (2.1 – 5.5) | 3.2 (2.0 – 5.1) | 3.1 (1.9 – 5.3) |
| Glaucoma present | 214 (4.6) | 110 (7.1) | 45 (2.9) | 59 (3.8) |
| Diabetic retinopathy present | 61 (1.3) | 25 (1.6) | 13 (0.8) | 23 (1.5) |
| Age-related macular degeneration present | 49 (1.2) | 18 (1.3) | 19 (1.4) | 12 (0.9) |
| Glaucoma medication use | 64 (1.4) | 45 (2.9) | 7 (0.4) | 12 (0.8) |
| Estimated glomerular filtration rate (mL/min/1.73m^2^) | 88.68 ± 14.42 | 87.30 ± 14.87 | 88.95 ± 14.17 | 89.98 ± 14.06 |
| Albuminuria (yes/no) | 343 (7.3) | 126 (8.1) | 103 (6.6) | 114 (7.3) |
| Major depressive episode (yes/no) | 149 (3.2) | 57 (3.6) | 42 (2.7) | 50 (3.2) |
| **MRI measures (outcome variables in additional analyses)** |  |  |  |  |
| Hippocampal volume (ml)* | 8,016.47 ± 804.36 | 7,894.19 ± 804.54 | 8,004.57 ± 786.72 | 8,150.12 ± 801.87 |
| Thalamus volume (ml)* | 13,527.88 ± 1,424.94 | 13,290.01 ± 1,419.64 | 13,481.72 ± 1,389.71 | 13,810.90 ± 1,417.59 |
| Cingulate area (mm^2^)* | 7,042.38 ± 916.67 | 7,021.52 ± 951.02 | 7,002.33 ± 871.88 | 7,103.24 ± 923.21 |
| Corpus callosum volume (ml)* | 3,352.51 ± 469.29 | 3,311.35 ± 484.70 | 3,357.06 ± 452.57 | 3,388.94 ± 467.31 |
| Cerebrellum volume (ml)* | 135,107.90 ± 13,237.07 | 134,068.98 ± 13,080.95 | 134,825.69 ± 13,132.65 | 136,424.74 ± 13,397.10 |
| Uncinate fasciculus volume (ml)* | 2,543,170.37 ± 498,468.67 | 2,502,689.32 ± 503,439.52 | 2,533,052.28 ± 484,799.02 | 2,593,804.88 ± 503,042.25 |

Supplemental Table S1 shows additional general study population characteristics according to tertiles of pRNFL thickness in the study population with complete data on cognitive performance.

Data are presented as mean ± standard deviation, median (interquartile range) or number (%).

* Data are available for (n=3,425).

Abbreviations: SD, standard deviation; pRNFL, peripapillary retinal nerve fiber layer; MRI, magnetic resonance imaging.

**Supplemental Table S2 Retinal and brain indices according to mild cognitive impairment status in the study population with complete data on pRNFL thickness and MRI data**

|  | With mild cognitive impairment | Without mild cognitive impairment |
| --- | --- | --- |
| Characteristic | N = 728 | N = 2,594 |
|  |  |  |
| **Determinants** |  |  |
| pRNFL (micrometer) | 94.89 ± 11.05 | 95.04 ± 10.94 |
| mRNFL (micrometer)* | 22.81 ± 6.97 | 22.57 ± 3.58 |
| mGCL(micrometer)* | 43.81 ± 4.59 | 44.18 ± 4.48 |
| mIPL (micrometer)* | 37.38 ± 3.19 | 37.64 ± 3.18 |
| **Brain** |  |  |
| Total grey matter brain volume (ml) | 47.61 ± 2.46 | 47.76 ± 2.28 |
| Total white matter brain volume (ml) | 33.89 ± 2.16 | 34.29 ± 2.01 |
| Whole brain node degree (edges) | 17.72 ± 0.37 | 17.79 ± 0.33 |
| Global efficiency (connections) | 0.84 ± 0.03 | 0.84 ± 0.03 |
| Clustering coefficient (no unit) | 2.32 ± 0.08 | 2.30 ± 0.07 |
| Local efficiency (connections) | 1.50 ± 0.04 | 1.49 ± 0.04 |

Supplemental Table S2 shows retinal and brain indices according to mild cognitive impairment status in the study population with complete data on pRNFL thickness and MRI data. Individuals with dementia (n=3) were not included.

Data are presented as mean ± standard deviation, median (interquartile range) or number (%).

* Data shown in the study population with complete data on mRNFL, mGCL mIPL, and MRI data (n=3,175; n=687 participants had mild cognitive impairment and n=2,488 participants did not have mild cognitive impairment).

Abbreviations: SD, standard deviation; pRNFL, peripapillary retinal nerve fiber layer; mRNFL, macular retinal nerve fiber layer; mGCL, macular ganglion cell layer; mIPL, macular inner plexiform layer; MRI, magnetic resonance imaging.

**Supplemental Table S3 General study population characteristics of the included and excluded participants for the study population with complete data on pRNFL, potential confounders, and cognitive performance**

| Characteristic | Included, N=4,697 | Excluded, N=2,992 | N missing data in excluded population |
| --- | --- | --- | --- |
| Age (years) | 59.27 ± 8.68 | 60.66 ± 8.64 | 0 |
| Sex |  |  | 0 |
| male | 2,257 (48) | 1,617 (54) |  |
| female | 2,440 (52) | 1,375 (46) |  |
| Educational status |  |  | 114 |
| Low | 1,591 (34) | 1,040 (36) |  |
| Middle | 1,343 (29) | 749 (26) |  |
| High | 1,763 (38) | 1,089 (38) |  |
| Glucose metabolism status |  |  | 0 |
| Normal glucose metabolism | 2,957 (63) | 1,648 (55) |  |
| Prediabetes | 712 (15) | 429 (14) |  |
| Type 2 diabetes | 1,008 (21) | 885 (30) |  |
| Other types of diabetes than type 2 | 20 (0.4) | 30 (1.0) |  |
| Spherical equivalent (dpt) | 0.06 (-1.25 – 1.06) | -0.06 (-1.88 – 1.06) | 626 |
| Office systolic blood pressure (mmHg) | 133.02 ± 17.63 | 134.97 ± 18.32 | 3 |
| Cardiovascular disease | 766 (16) | 533 (18) | 98 |
| Waist circumference (cm) | 94.37 ± 13.28 | 96.86 ± 14.24 | 5 |
| Total/HDL cholesterol ratio (no unit) | 3.36 (2.75 – 4.17) | 3.50 (2.82 – 4.33) | 6 |
| Use of lipid-modifying medication | 1,416 (30) | 1,088 (36) | 6 |
| Use of antihypertensive medication | 1,681 (36) | 1,255 (42) | 6 |
| Alcohol consumption |  |  | 64 |
| None | 830 (18) | 583 (20) |  |
| Low | 2,743 (58) | 1,688 (58) |  |
| High | 1,124 (24) | 657 (22) |  |
| Smoking status |  |  | 63 |
| Never | 1,820 (39) | 1,015 (35) |  |
| Former | 2,293 (49) | 1,471 (50) |  |
| Current | 584 (12) | 443 (15) |  |
| **Retinal thickness indices (determinants)** |  |  |  |
| -pRNFL thickness (micrometers) | 94.92 ± 10.89 | 94.65 ± 10.59 | 2,338 |
| -mRNFL thickness (micrometers)* | 22.53 ± 4.23 | 22.42 ± 3.67 | 2,656 |
| -mGCL thickness (micrometers)* | 43.99 ± 4.54 | 43.60 ± 4.86 | 2,656 |
| -mIPL thickness (micrometers)* | 37.50 ± 3.17 | 37.31 ± 3.35 | 2,656 |
| **Cognitive performance (outcomes)** |  |  |  |
| Global cognitive performance (SD)** | 0.05 ± 0.67 | -0.05 ± 0.69 | 473 |

Supplemental Table S3 shows general study population characteristics of the included and excluded participants for the study population with complete data on pRNFL, potential confounders, and cognitive performance.

Data are presented as mean ± standard deviation, median (interquartile range) or number (%).

* Shown for study population with complete data on retinal macular layers.

**Global cognitive performance was standardized in the study population with complete data on global cognitive performance (n=7,216)

Abbreviations: SD, standard deviation; pRNFL, peripapillary retinal nerve fiber layer; mRNFL, macular retinal nerve fiber layer; mGCL, macular ganglion cell layer; mIPL, macular inner plexiform layer; HDL, high-density lipid.

**Supplemental Table S4 General study population characteristics of the included and excluded participants for the study population with complete data on pRNFL, potential confounders, and MRI measures**

| Characteristic | Included, N=3,436 | Excluded, N=4,253 | N missing data in excluded population |
| --- | --- | --- | --- |
| Age (years) | 58.99 ± 8.69 | 60.47 ± 8.63 | 0 |
| Sex |  |  | 0 |
| male | 1,675 (49) | 2,199 (52) |  |
| female | 1,761 (51) | 2,054 (48) |  |
| Educational status |  |  | 114 |
| Low | 1,123 (33) | 1,508 (36) |  |
| Middle | 1,005 (29) | 1,087 (26) |  |
| High | 1,308 (38) | 1,544 (37) |  |
| Glucose metabolism status |  |  | 0 |
| Normal glucose metabolism | 2,258 (66) | 2,347 (55) |  |
| Prediabetes | 513 (15) | 628 (15) |  |
| Type 2 diabetes | 650 (19) | 1,243 (29) |  |
| Type 1 and other diabetes | 15 (0.4) | 35 (0.8) |  |
| Spherical equivalent (dpt) | 0.06 (-1.38 – 1.06) | 0.06 (-1.50 – 1.09) | 626 |
| Office systolic blood pressure (mmHg) | 132.41 ± 17.18 | 134.88 ± 18.43 | 3 |
| Cardiovascular disease | 426 (12) | 873 (21) | 98 |
| Waist circumference (cm) | 93.37 ± 12.66 | 96.94 ± 14.32 | 5 |
| Total/HDL cholesterol ratio (no unit) | 3.36 (2.75 – 4.16) | 3.47 (2.81 – 4.31) | 6 |
| Use of lipid-modifying medication | 904 (26) | 1,600 (38) | 6 |
| Use of antihypertensive medication | 1,114 (32) | 1,822 (43) | 6 |
| Alcohol consumption (yes/no) |  |  | 64 |
| None | 587 (17) | 826 (20) |  |
| Low | 2,015 (59) | 2,416 (58) |  |
| High | 834 (24) | 947 (23) |  |
| Smoking status |  |  | 63 |
| Never | 1,387 (40) | 1,448 (35) |  |
| Former | 1,646 (48) | 2,118 (51) |  |
| Current | 403 (12) | 624 (15) |  |
| MRI lagtime | 0.69 (0.26 – 1.09) | 0.90 (0.47 – 3.04) | 2,506 |
| **Retinal thickness (determinants)** |  |  |  |
| -pRNFL thickness (micrometers) | 95.04 ± 10.98 | 94.62 ± 10.61 | 2,338 |
| -mRNFL thickness (micrometers)* | 22.53 ± 4.24 | 22.52 ± 4.20 | 2,656 |
| -mGCL thickness (micrometers)* | 44.26 ± 4.38 | 43.59 ± 4.81 | 2,656 |
| -mIPL thickness (micrometers)* | 37.68 ± 3.09 | 37.25 ± 3.33 | 2,656 |
| **MRI measures (outcomes)** |  |  |  |
| Grey matter (%) | 47.73 ± 2.32 | 47.42 ± 2.44 | 2,506 |
| White matter (%) | 34.20 ± 2.06 | 34.10 ± 2.12 | 2,506 |
| Whole brain node degree (edges) | 17.77 ± 0.34 | 17.72 ± 0.39 | 2,542 |
| Global efficiency (connections) | 0.84 ± 0.03 | 0.83 ± 0.03 | 2,542 |
| Clustering coefficient (no unit) | 2.31 ± 0.08 | 2.32 ± 0.09 | 2,542 |
| Local efficiency (connections) | 1.49 ± 0.04 | 1.50 ± 0.04 | 2,542 |

Supplemental Table S4 shows general study population characteristics of the included and excluded participants for the study population with complete data on pRNFL, potential confounders, and MRI measures.

Data are presented as mean ± standard deviation, median (interquartile range) or number (%).

* Shown for study population with complete data on retinal macular layers.

Abbreviations: SD, standard deviation; pRNFL, peripapillary retinal nerve fiber layer; mRNFL, macular retinal nerve fiber layer; mGCL, macular ganglion cell layer; mIPL, macular inner plexiform layer; HDL, high-density lipid.

**Supplemental Table S5 P-values for interaction by sex, prediabetes, and type 2 diabetes in the associations of retinal thickness indices with cognitive performance and brain MRI measures**

|  | **Cognitive performance** | **Total grey matter volume** | **Total white matter volume** | **Whole brain node degree** | **Global efficiency** | **Clustering coefficient** | **Local efficiency** |
| --- | --- | --- | --- | --- | --- | --- | --- |
|  | P-value | P-value | P-value | P-value | P-value | P-value | P-value |
| pRNFL | | | | |  |  |  |
| -Sex | 0.64 | 0.89 | 0.64 | 0.67 | 0.053 | 0.90 | 0.98 |
| -Prediabetes | 0.27 | 0.22 | 0.84 | 0.55 | 0.086 | 0.39 | 0.43 |
| -Type 2 diabetes | 0.38 | 0.23 | 0.18 | 0.35 | 0.66 | 0.30 | 0.29 |
| mRNFL |  |  |  |  |  |  |  |
| -Sex | 0.56 | 0.40 | 0.051 | 0.47 | 0.54 | 0.18 | 0.17 |
| -Prediabetes | 0.60 | 0.30 | 0.14 | 0.22 | >0.99 | 0.48 | 0.60 |
| -Type 2 diabetes | 0.80 | 0.63 | **0.012** | 0.08 | 0.66 | 0.46 | 0.58 |
| mGCL |  |  |  |  |  |  |  |
| -Sex | 0.97 | 0.37 | 0.065 | 0.23 | **0.005** | 0.12 | 0.10 |
| -Prediabetes | 0.63 | 0.33 | **0.021** | 0.32 | 0.77 | 0.76 | 0.90 |
| -Type 2 diabetes | 0.62 | 0.37 | 0.24 | 0.39 | 0.12 | 0.36 | 0.49 |
| mIPL |  |  |  |  |  |  |  |
| -Sex | 0.95 | 0.39 | 0.12 | 0.13 | **0.016** | 0.076 | 0.06 |
| -Prediabetes | 0.36 | 0.33 | 0.14 | 0.83 | 0.82 | 0.61 | 0.52 |
| -Type 2 diabetes | 0.46 | 0.46 | 0.58 | 0.84 | 0.12 | 0.68 | 0.89 |

Supplemental Table S5 shows P-values for interaction by sex, prediabetes, and type 2 diabetes in the associations of retinal thickness indices with cognitive performance and brain MRI measures. P-values represent the P-values for the interaction terms of sex, glucose metabolism status (i.e. prediabetes versus normal glucose metabolism status or type 2 diabetes versus normal glucose metabolism status) with determinants (e.g., sex*pRNFL) in the associations of retinal thickness indices with cognitive performance and MRI measures. Variables in the model: age, sex, educational status, spherical equivalent, office systolic blood pressure, total cholesterol/HDL cholesterol ratio, use of antihypertensive or lipid-modifying medication, waist circumference, MRI lag time (only applicable for MRI measures), smoking status, and alcohol consumption status. We entered interaction terms for all covariates in the model. In addition, for interaction analyses with glucose metabolism status, we did not include individuals with other types of diabetes than type 2 diabetes..

P-value <0.05 denotes statistically significant interaction.

Abbreviations: pRNFL, peripapillary retinal nerve fiber layer thickness; mRNFL, macular retinal nerve fiber layer thickness; mGCL, macular ganglion cell layer thickness; mIPL, macular inner plexiform layer; MRI, magnetic resonance imaging; HDL, high-density lipid.

**Supplemental Table S6 Associations of retinal thickness indices with memory, executive function, and information processing speed**

| **Retinal thickness indices** | **Model** | **N** | **Memory** | | **Executive function** | | **Information processing speed** | |
| --- | --- | --- | --- | --- | --- | --- | --- | --- |
|  |  |  | Beta (95% CI) | P-value | Beta (95% CI) | P-value | Beta (95% CI) | P-value |
| Peripapillary retinal nerve fiber layer thickness, low versus high | Crude | 4,697 | **-0.10 (-0.16 to -0.03)** | **0.004** | -0.06 (-0.13 to 0.00) | 0.057 | **-0.09 (-0.16 to -0.03)** | **0.006** |
|  | 1 | 4,697 | -0.02 (-0.08 to 0.04) | 0.52 | -0.05 (-0.11 to 0.01) | 0.13 | **-0.06 (-0.11 to 0.00)** | **0.047** |
|  | 2 | 4,697 | -0.02 (-0.08 to 0.04) | 0.55 | -0.05 (-0.11 to 0.02) | 0.14 | **-0.06 (-0.12 to 0.00)** | **0.041** |
| Macular retinal nerve fiber layer thickness, low versus high | Crude | 4,340 | -0.04 (-0.11 to 0.03) | 0.23 | **-0.12 (-0.19 to -0.06)** | **<0.001** | **-0.16 (-0.23 to -0.09)** | **<0.001** |
|  | 1 | 4,340 | -0.03 (-0.09 to 0.03) | 0.35 | -0.04 (-0.11 to 0.02) | 0.18 | **-0.08 (-0.14 to -0.02)** | **0.009** |
|  | 2 | 4,340 | -0.02 (-0.08 to 0.04) | 0.46 | -0.04 (-0.10 to 0.02) | 0.22 | **-0.07 (-0.13 to -0.01)** | **0.019** |
| Macular ganglion cell layer thickness, per SD lower | Crude | 4,340 | **-0.08 (-0.11 to -0.05)** | **<0.001** | **-0.11 (-0.14 to -0.09)** | **<0.001** | **-0.14 (-0.17 to -0.11)** | **<0.001** |
|  | 1 | 4,340 | -0.02 (-0.04 to 0.01) | 0.18 | **-0.03 (-0.06 to 0.00)** | **0.024** | **-0.04 (-0.07 to -0.02)** | **<0.001** |
|  | 2 | 4,340 | -0.01 (-0.04 to 0.01) | 0.27 | **-0.03 (-0.06 to 0.00)** | **0.034** | **-0.04 (-0.06 to -0.01)** | **0.002** |
| Macular inner plexiform layer thickness, per SD lower | Crude | 4,340 | **-0.07 (-0.10 to -0.04)** | **<0.001** | **-0.11 (-0.14 to -0.08)** | **<0.001** | **-0.13 (-0.16 to -0.10)** | **<0.001** |
|  | 1 | 4,340 | -0.02 (-0.05 to 0.00) | 0.086 | **-0.03 (-0.05 to -0.01)** | **0.016** | **-0.03 (-0.05 to -0.01)** | **0.004** |
|  | 2 | 4,340 | -0.02 (-0.05 to 0.01) | 0.12 | **-0.03 (-0.06 to 0.00)** | **0.021** | **-0.03 (-0.06 to -0.01)** | **0.011** |

Supplemental Table S6 shows the associations of retinal thickness indices with memory, executive function, and information processing speed. Values per SD or quartile of retinal thickness indices are shown in the legend of Table 2 in the main manuscript.

Variables entered in models: Crude: none; Model 1: + age, sex, glucose metabolism status, educational level, spherical equivalent; Model 2: model 1 + office systolic blood pressure, history of cardiovascular disease, waist circumference, smoking status, alcohol consumption, Total/HDL cholesterol ratio, lipid-modifying medication, and antihypertensive medication.

Bold denotes P<0.05.

Abbreviations: CI, confidence interval; SD, standard deviation; N, population sample size.

**Supplemental Table S7 Associations of retinal thickness indices with global cognitive performance, with additional adjustment for diet and physical activity (model 3A); the presence of a major depressive episode (model 3B); biomarkers of inflammation (model 3C); ocular pathology (diabetic retinopathy, glaucoma, age-related macular degeneration; model 3D); kidney variables (i.e. eGFR, albuminuria; model 3E); or after exclusion of individuals with ocular pathology (i.e. diabetic retinopathy, glaucoma, and/or age-related macular degeneration; model 3F)**

| **Retinal thickness indices** | **Model** | **N** | **Global cognitive performance** | |
| --- | --- | --- | --- | --- |
|  |  |  | Beta (95% CI) | P-value |
| Peripapillary retinal nerve fiber layer thickness, low versus high | 3A | 3,841 | **-0.06 (-0.12 to 0.001)** | **0.038** |
|  | 3B | 4,676 | -0.05 (-0.10 to 0.00) | 0.061 |
|  | 3C | 2,093 | -0.06 (-0.15 to 0.02) | 0.13 |
|  | 3D | 4,009 | -0.05 (-0.10 to 0.01) | 0.13 |
|  | 3E | 1,892 | -0.06 (-0.14 to 0.03) | 0.21 |
|  | 3F | 3,742 | -0.03 (-0.09 to 0.03) | 0.35 |
| Macular retinal nerve fiber layer thickness, low versus high | 3A | 3,860 | -0.05 (-0.11 to 0.01) | 0.14 |
|  | 3B | 4,321 | -0.05 (-0.10 to 0.001) | 0.051 |
|  | 3C | 2,357 | -0.07 (-0.14 to 0.01) | 0.074 |
|  | 3D | 4,147 | **-0.06 (-0.12 to -0.001)** | **0.034** |
|  | 3E | 2,132 | -0.05 (-0.13 to 0.03) | 0.24 |
|  | 3F | 3,444 | -0.06 (-0.12 to 0.01) | 0.075 |
| Macular ganglion cell layer thickness, per SD lower | 3A | 3,520 | **-0.03 (-0.06 to -0.01)** | **0.013** |
|  | 3B | 4,321 | **-0.03 (-0.06 to -0.01)** | **0.007** |
|  | 3C | 2,357 | **-0.06 (-0.09 to -0.03)** | **<0.001** |
|  | 3D | 4,147 | **-0.03 (-0.06 to -0.01)** | **0.005** |
|  | 3E | 2,132 | **-0.06 (-0.09 to -0.02)** | **<0.001** |
|  | 3F | 3,444 | **-0.04 (-0.06 to -0.01)** | **0.005** |
| Macular inner plexiform layer thickness, per SD lower | 3A | 3,520 | **-0.03 (-0.06 to -0.01)** | **0.011** |
|  | 3B | 4,321 | **-0.03 (-0.06 to -0.01)** | **0.004** |
|  | 3C | 2,357 | **-0.07 (-0.10 to -0.03)** | **<0.001** |
|  | 3D | 4,147 | **-0.04 (-0.06 to -0.01)** | **0.003** |
|  | 3E | 2,132 | **-0.07 (-0.10 to -0.03)** | **<0.001** |
|  | 3F | 3,444 | **-0.04 (-0.07 to -0.02)** | **0.002** |

Supplemental Table S7 shows the associations of retinal thickness indices with cognitive performance. Of note, non-linear associations are shown for macular and peripapillary retinal nerve fiber layer thickness. For retinal indices, values per SD or quartile are numerically similar to the values reported in the legend of Table 2 of the main manuscript.

Variables entered in all models: age, sex, glucose metabolism status, educational level, spherical equivalent, office systolic blood pressure, history of cardiovascular disease, waist circumference, smoking status, alcohol consumption, Total/HDL cholesterol ratio, lipid-modifying medication, and antihypertensive medication.

Bold denotes P<0.05.

Abbreviations: CI, confidence interval; SD, standard deviation; N, population sample size; HDL, high-density lipid.

**Supplemental Table S8 Associations of retinal thickness indices with MRI measures, with additional adjustment for diet and physical activity (model 3A); biomarkers of inflammation (model 3B); ocular pathology (diabetic retinopathy, glaucoma, age-related macular degeneration; 3C); kidney variables (i.e. eGFR, albuminuria; model 3D); or after exclusion of individuals with ocular pathology (i.e. diabetic retinopathy, glaucoma, and/or age-related macular degeneration; model 3E)**

| **Retinal thickness indices** | | | | **Total grey matter volume** | | **Total white matter volume** | | **Whole brain node degree** | | **Global efficiency** | | **Clustering coefficient** | | **Local efficiency** | |
| --- | --- | --- | --- | --- | --- | --- | --- | --- | --- | --- | --- | --- | --- | --- | --- |
|  | Model | Number of participants |  | |  |  |  |  |  |  |  |  |  |  |  |
| Peripapillary retinal nerve fiber layer thickness, per SD lower | 3A | 2,818 | 0.00 (-0.03 to 0.03) | | >0.99 | **-0.12 (-0.16 to -0.08)** | **<0.001** | **-0.09 (-0.13 to -0.06)** | **<0.001** | **-0.05 (-0.09 to -0.01)** | **0.015** | **0.04 (0.001 to 0.08)** | **0.044** | **0.04 (0.01 to 0.08)** | **0.021** |
|  | 3B | 1,571 | -0.01 (-0.06 to 0.04) | | 0.69 | **-0.09 (-0.14 to -0.04)** | **<0.001** | **-0.08 (-0.13 to -0.02)** | **0.004** | -0.03 (-0.08 to 0.02) | 0.29 | 0.03 (-0.02 to 0.08) | 0.19 | 0.04 (-0.01 to 0.09) | 0.15 |
|  | 3C | 2,964 | 0.01 (-0.02 to 0.05) | | 0.43 | **-0.13 (-0.17 to -0.09)** | **<0.001** | **-0.12 (-0.16 to -0.08)** | **<0.001** | **-0.04 (-0.08 to 0.00)** | **0.030** | **0.06 (0.02 to 0.10)** | **0.001** | **0.07 (0.03 to 0.10)** | **<0.001** |
|  | 3D | 1,410 | -0.01 (-0.06 to 0.04) | | 0.61 | **-0.09 (-0.15 to -0.04)** | **<0.001** | **-0.07 (-0.12 to -0.01)** | **0.012** | -0.03 (-0.09 to 0.03) | 0.32 | 0.03 (-0.03 to 0.08) | 0.30 | 0.03 (-0.03 to 0.08) | 0.30 |
|  | 3E | 2,782 | 0.01 (-0.03 to 0.04) | | 0.75 | **-0.12 (-0.16 to -0.08)** | **<0.001** | **-0.11 (-0.15 to -0.07)** | **<0.001** | -0.04 (-0.08 to 0.00) | 0.083 | **0.06 (0.02 to 0.10)** | **0.002** | **0.06 (0.02 to 0.10)** | **0.001** |
| Macular retinal nerve fiber layer thickness, per SD lower | 3A | 2,671 | 0.01 (-0.02 to 0.04) | | 0.52 | -0.02 (-0.06 to 0.01) | 0.23 | 0.01 (-0.03 to 0.05) | 0.55 | 0.00 (-0.04 to 0.04) | 0.93 | -0.02 (-0.06 to 0.01) | 0.24 | -0.02 (-0.06 to 0.01) | 0.20 |
|  | 3B | 1,747 | -0.01 (-0.05 to 0.03) | | 0.69 | -0.01 (-0.05 to 0.04) | 0.71 | 0.00 (-0.05 to 0.04) | 0.90 | 0.00 (-0.05 to 0.05) | 0.98 | -0.02 (-0.07 to 0.02) | 0.29 | -0.03 (-0.07 to 0.02) | 0.28 |
|  | 3C | 2,817 | 0.01 (-0.02 to 0.05) | | 0.46 | **-0.04 (-0.08 to -0.001)** | **0.027** | -0.04 (-0.08 to 0.001) | 0.058 | -0.01 (-0.05 to 0.02) | 0.47 | 0.02 (-0.02 to 0.06) | 0.33 | 0.02 (-0.01 to 0.06) | 0.22 |
|  | 3D | 1,563 | -0.01 (-0.06 to 0.03) | | 0.51 | -0.01 (-0.05 to 0.04) | 0.82 | -0.01 (-0.05 to 0.04) | 0.83 | 0.00 (-0.05 to 0.05) | 0.86 | -0.03 (-0.08 to 0.01) | 0.16 | -0.03 (-0.08 to 0.01) | 0.16 |
|  | 3E | 2,632 | 0.01 (-0.02 to 0.04) | | 0.55 | **-0.05 (-0.09 to -0.02)** | **0.005** | -0.03 (-0.06 to 0.01) | 0.19 | -0.01 (-0.05 to 0.03) | 0.76 | -0.01 (-0.05 to 0.03) | 0.68 | 0.00 (-0.04 to 0.04) | 0.96 |
| Macular ganglion cell layer thickness, per SD lower | 3A | 2,671 | **-0.04 (-0.08 to -0.01)** | | **0.013** | **-0.07 (-0.11 to -0.03)** | **<0.001** | **-0.11 (-0.15 to -0.07)** | **<0.001** | -0.03 (-0.07 to 0.01) | 0.10 | **0.07 (0.03 to 0.11)** | **<0.001** | **0.07 (0.04 to 0.11)** | **<0.001** |
|  | 3B | 1,747 | **-0.04 (-0.09 to -0.001)** | | **0.038** | -0.02 (-0.07 to 0.02) | 0.36 | **-0.12 (-0.16 to -0.07)** | **<0.001** | -0.04 (-0.09 to 0.01) | 0.09 | **0.06 (0.01 to 0.10)** | **0.013** | **0.07 (0.02 to 0.11)** | **0.004** |
|  | 3C | 2,817 | **-0.04 (-0.07 to -0.01)** | | **0.018** | **-0.09 (-0.12 to -0.05)** | **<0.001** | **-0.13 (-0.17 to -0.10)** | **<0.001** | -0.03 (-0.07 to 0.01) | 0.14 | **0.08 (0.05 to 0.12)** | **<0.001** | **0.09 (0.06 to 0.13)** | **<0.001** |
|  | 3D | 1,563 | **-0.05 (-0.09 to -0.01)** | | **0.026** | -0.03 (-0.08 to 0.02) | 0.26 | **-0.13 (-0.18 to -0.08)** | **<0.001** | -0.04 (-0.09 to 0.01) | 0.14 | **0.06 (0.02 to 0.11)** | **0.009** | **0.07 (0.02 to 0.12)** | **0.003** |
|  | 3E | 2,632 | **-0.05 (-0.08 to -0.01)** | | **0.008** | **-0.07 (-0.11 to -0.03)** | **<0.001** | **-0.12 (-0.16 to -0.08)** | **<0.001** | -0.03 (-0.07 to 0.01) | 0.16 | **0.07 (0.03 to 0.10)** | **<0.001** | **0.08 (0.04 to 0.11)** | **<0.001** |
| Macular inner plexiform layer thickness, per SD lower | 3A | 2,671 | **-0.04 (-0.07 to -0.001)** | | **0.026** | **-0.07 (-0.10 to -0.03)** | **<0.001** | **-0.10 (-0.14 to -0.06)** | **<0.001** | -0.02 (-0.06 to 0.01) | 0.34 | **0.06 (0.02 to 0.10)** | **0.001** | **0.07 (0.03 to 0.10)** | **<0.001** |
|  | 3B | 1,747 | -0.04 (-0.08 to 0.001) | | 0.079 | -0.03 (-0.07 to 0.02) | 0.29 | **-0.11 (-0.16 to -0.07)** | **<0.001** | -0.03 (-0.08 to 0.02) | 0.23 | **0.05 (0.001 to 0.10)** | **0.036** | **0.06 (0.01 to 0.11)** | **0.013** |
|  | 3C | 2,817 | **-0.04 (-0.07 to -0.01)** | | **0.024** | **-0.09 (-0.12 to -0.05)** | **<0.001** | **-0.13 (-0.16 to -0.09)** | **<0.001** | -0.02 (-0.06 to 0.02) | 0.37 | **0.08 (0.04 to 0.11)** | **<0.001** | **0.09 (0.05 to 0.12)** | **<0.001** |
|  | 3D | 1,563 | -0.04 (-0.09 to 0.001) | | 0.053 | -0.03 (-0.08 to 0.02) | 0.23 | **-0.13 (-0.17 to -0.08)** | **<0.001** | -0.03 (-0.08 to 0.02) | 0.31 | **0.06 (0.01 to 0.11)** | **0.016** | **0.07 (0.02 to 0.12)** | **0.005** |
|  | 3E | 2,632 | **-0.04 (-0.08 to -0.01)** | | **0.015** | **-0.08 (-0.11 to -0.04)** | **<0.001** | **-0.12 (-0.15 to -0.08)** | **<0.001** | -0.02 (-0.06 to 0.02) | 0.40 | **0.06 (0.03 to 0.10)** | **<0.001** | **0.07 (0.04 to 0.11)** | **<0.001** |

Supplemental Table S8 shows the associations of retinal thickness indices with MRI measures Values per SD or quartile or retinal thickness are numerically similar to the values reported in the legend of Table 2 of the main manuscript; and values per SD of brain MRI measures are numerically similar to the values reported in the legends of Table 3 and 4.

Variables entered in all models: age, sex, glucose metabolism status, educational level, MRI lag time, spherical equivalent, office systolic blood pressure, history of cardiovascular disease, waist circumference, smoking status, alcohol consumption, Total/HDL cholesterol ratio, lipid-modifying medication, and antihypertensive medication.

Bold denotes P<0.05.

Abbreviations: CI, confidence interval; SD, standard deviation; N, population sample size; HDL, high-density lipid; MRI, magnetic resonance imaging.
